# Subgradient ellipsoid method for nonsmooth convex problems

**DOI:** 10.1007/s10107-022-01833-4

**Published:** 2022-06-14

**Authors:** Anton Rodomanov, Yurii Nesterov

**Affiliations:** 1 Institute of Information and Communication Technologies, Electronics and Applied Mathematics (ICTEAM), Catholic University of Louvain (UCL), Louvain-la-Neuve, Belgium; 2 Center for Operations Research and Econometrics (CORE), Catholic University of Louvain (UCL), Louvain-la-Neuve, Belgium

**Keywords:** Subgradient method, Ellipsoid method, Accuracy certificates, Separating oracle, Convex optimization, Nonsmooth optimization, Saddle-point problems, Variational inequalities, 90C25, 90C47, 68Q25

## Abstract

In this paper, we present a new ellipsoid-type algorithm for solving nonsmooth problems with convex structure. Examples of such problems include nonsmooth convex minimization problems, convex-concave saddle-point problems and variational inequalities with monotone operator. Our algorithm can be seen as a combination of the standard Subgradient and Ellipsoid methods. However, in contrast to the latter one, the proposed method has a reasonable convergence rate even when the dimensionality of the problem is sufficiently large. For generating accuracy certificates in our algorithm, we propose an efficient technique, which ameliorates the previously known recipes (Nemirovski in Math Oper Res 35(1):52–78, 2010).

## Introduction

The Ellipsoid Method is a classical algorithm in Convex Optimization. It was proposed in 1976 by Yudin and Nemirovski [23] as the modified method of centered cross-sections and then independently rediscovered a year later by Shor [21] in the form of the subgradient method with space dilation. However, the popularity came to the Ellipsoid Method only when Khachiyan used it in 1979 for proving his famous result on polynomial solvability of Linear Programming [10]. Shortly after, several polynomial algorithms, based on the Ellipsoid Method, were developed for some combinatorial optimization problems [9]. For more details and historical remarks on the Ellipsoid Method, see [2, 3, 14].

Despite its long history, the Ellipsoid Method still has some issues which have not been fully resolved or have been resolved only recently. One of them is the computation of accuracy certificates which is important for generating approximate solutions to dual problems or for solving general problems with convex structure (saddle-point problems, variational inequalities, etc.). For a long time, the procedure for calculating an accuracy certificate in the Ellipsoid Method required solving an auxiliary piecewise linear optimization problem (see, e.g., sect. 5 and 6 in [14]). Although this auxiliary computation did not use any additional calls to the oracle, it was still computationally expensive and, in some cases, could take even more time than the Ellipsoid Method itself. Only recently an efficient alternative has been proposed [16].

Another issue with the Ellipsoid Method is related to its poor dependency on the dimensionality of the problem. Consider, e.g., the minimization problem

1 $$\begin{aligned} \min _{x \in Q} f(x), \end{aligned}$$

where $f :\mathbb {R}^n \rightarrow \mathbb {R}$ is a convex function and $Q :=\lbrace {x \in \mathbb {R}^n : \Vert x \Vert \le R} \rbrace$ is the Euclidean ball of radius $R > 0$. The Ellipsoid Method for solving (1) can be written as follows (see, e.g., sect. 3.2.8 in [19]):

2 $$\begin{aligned} \begin{aligned} x_{k+1}&:=x_k - \frac{1}{n + 1} \frac{W_k g_k}{\langle {g_k, W_k g_k} \rangle ^{1/2}}, \\ W_{k+1}&:=\frac{n^2}{n^2 - 1} \biggl ( W_k - \frac{2}{n + 1} \frac{W_k g_k g_k^TW_k}{\langle {g_k, W_k g_k} \rangle } \biggr ), \qquad k \ge 0, \end{aligned} \end{aligned}$$

where $x_0 :=0$, $W_0 :=R^2 I$ (*I* is the identity matrix) and $g_k :=f'(x_k)$ is an arbitrary nonzero subgradient if $x_k \in Q$, and $g_k$ is an arbitrary separator^1^ of $x_k$ from *Q* if $x_k \notin Q$.

To solve problem (1) with accuracy $\epsilon > 0$ (in terms of the function value), the Ellipsoid Method needs

3 $$\begin{aligned} O\Bigl ( n^2 \ln \frac{M R}{\epsilon } \Bigr ) \end{aligned}$$

iterations, where $M > 0$ is the Lipschitz constant of *f* on *Q* (see theorem 3.2.11 in [19]). Looking at this estimate, we can see an immediate drawback: it directly depends on the dimension and becomes useless when $n \rightarrow \infty$. In particular, we cannot guarantee any reasonable rate of convergence for the Ellipsoid Method when the dimensionality of the problem is sufficiently big.

Note that the aforementioned drawback is an artifact of the method itself, not its analysis. Indeed, when $n \rightarrow \infty$, iteration (2) reads

$$\begin{aligned} x_{k+1} :=x_k, \quad W_{k+1} :=W_k, \quad k \ge 0. \end{aligned}$$

Thus, the method stays at the same point and does not make any progress.

On the other hand, the simplest Subgradient Method for solving (1) possesses the “dimension-independent” $O(M^2 R^2 / \epsilon ^2)$ iteration complexity bound (see, e.g., sect. 3.2.3 in [19]). Comparing this estimate with (3), we see that the Ellipsoid Method is significantly faster than the Subgradient Method only when *n* is not too big compared to $M R / \epsilon$ and significantly slower otherwise. Clearly, this situation is strange because the former algorithm does much more work at every iteration by “improving” the “metric” $W_k$ which is used for measuring the norm of the subgradients.

In this paper, we propose a new ellipsoid-type algorithm for solving nonsmooth problems with convex structure, which does not have the discussed above drawback. Our algorithm can be seen as a combination of the Subgradient and Ellipsoid methods and its convergence rate is basically as good as the best of the corresponding rates of these two methods (up to some logarithmic factors). In particular, when $n \rightarrow \infty$, the convergence rate of our algorithm coincides with that of the Subgradient Method.

### Contents

This paper is organized as follows. In Sect. 2.1, we review the general formulation of a problem with convex structure and the associated with it notions of *accuracy certificate* and *residual*. Our presentation mostly follows [16] with examples taken from [18]. Then, in Sect. 2.2, we introduce the notions of *accuracy semicertificate* and *gap* and discuss their relation with those of accuracy certificate and residual.

In Sect. 3, we present the general algorithmic scheme of our methods. To measure the convergence rate of this scheme, we introduce the notion of *sliding gap* and establish some preliminary bounds on it.

In Sect. 4, we discuss different choices of parameters in our general scheme. First, we show that, by setting some of the parameters to zero, we obtain the standard Subgradient and Ellipsoid methods. Then we consider a couple of other less trivial choices which lead to two new algorithms. The principal of these new algorithms is the latter one, which we call the *Subgradient Ellipsoid Method*. We demonstrate that the convergence rate of this algorithm is basically as good as the best of those of the Subgradient and Ellipsoid methods.

In Sect. 5, we show that, for both our new methods, it is possible to efficiently generate accuracy semicertificates whose gap is upper bounded by the sliding gap. We also compare our approach with the recently proposed technique from [16] for building accuracy certificates for the standard Ellipsoid Method.

In Sect. 6, we discuss how to efficiently implement our general scheme and the procedure for generating accuracy semicertificates. In particular, we show that the time and memory requirements of our scheme are the same as in the standard Ellipsoid Method.

Finally, in Sect. 7, we discuss some open questions.

### Notation and generalities

In this paper, $\mathbb {E}$ denotes an arbitrary *n*-dimensional real vector space. Its dual space, composed of all linear functionals on $\mathbb {E}$, is denoted by $\mathbb {E}^*$. The value of $s \in \mathbb {E}^*$, evaluated at $x \in \mathbb {E}$, is denoted by $\langle {s, x} \rangle$. See [19, sect. 4.2.1] for the supporting discussion of abstract real vector spaces in Optimization.

Let us introduce in the spaces $\mathbb {E}$ and $\mathbb {E}^*$ a pair of conjugate Euclidean norms. To this end, let us fix a self-adjoint positive definite linear operator $B :\mathbb {E} \rightarrow \mathbb {E}^*$ and define

$$\begin{aligned} \Vert x \Vert :=\langle {B x, x} \rangle ^{1/2}, \quad x \in \mathbb {E}, \qquad \quad {\Vert {s} \Vert }_* :=\langle {s, B^{-1} s} \rangle ^{1/2}, \quad s \in \mathbb {E}^*. \end{aligned}$$

Note that, for any $s \in \mathbb {E}^*$ and $x \in \mathbb {E}$, we have the Cauchy-Schwarz inequality

$$\begin{aligned} |\langle {s, x} \rangle | \le {\Vert {s} \Vert }_* \Vert x \Vert , \end{aligned}$$

which becomes an equality if and only if *s* and *Bx* are collinear. In addition to $\Vert x \Vert$ and ${\Vert {\cdot } \Vert }_*$, we often work with other Euclidean norms defined in the same way but using another reference operator instead of *B*. In this case, we write ${\Vert \cdot \Vert }_{G}$ and ${\Vert \cdot \Vert }_{G}^*$, where $G :\mathbb {E} \rightarrow \mathbb {E}^*$ is the corresponding self-adjoint positive definite linear operator.

Sometimes, in the formulas, involving products of linear operators, it is convenient to treat $x \in \mathbb {E}$ as a linear operator from $\mathbb {R}$ to $\mathbb {E}$, defined by $x \alpha :=\alpha x$, and $x^*$ as a linear operator from $\mathbb {E}^*$ to $\mathbb {R}$, defined by $x^*s :=\langle {s, x} \rangle$. Likewise, any $s \in \mathbb {E}^*$ can be treated as a linear operator from $\mathbb {R}$ to $\mathbb {E}^*$, defined by $s \alpha :=\alpha s$, and $s^*$ as a linear operator from $\mathbb {E}$ to $\mathbb {R}$, defined by $s^*x :=\langle {s, x} \rangle$. Then, $x x^*$ and $s s^*$ are rank-one self-adjoint linear operators from $\mathbb {E}^*$ to $\mathbb {E}$ and from $\mathbb {E}$ to $\mathbb {E}^*$ respectively, acting as follows: $(x x^*) s = \langle {s, x} \rangle x$ and $(s s^*) x = \langle {s, x} \rangle s$ for any $x \in \mathbb {E}$ and $s \in \mathbb {E}^*$.

For a self-adjoint linear operator $G :\mathbb {E} \rightarrow \mathbb {E}^*$, by ${{\,\mathrm{tr}\,}}G$ and $\det G$, we denote the trace and determinant of *G* with respect to our fixed operator *B*:

$$\begin{aligned} {{\,\mathrm{tr}\,}}G :={{\,\mathrm{tr}\,}}(B^{-1} G), \qquad \det G :=\det (B^{-1} G). \end{aligned}$$

Note that, in these definitions, $B^{-1} G$ is a linear operator from $\mathbb {E}$ to $\mathbb {E}$, so ${{\,\mathrm{tr}\,}}(B^{-1} G)$ and $\det (B^{-1} G)$ are the standard well-defined notions of trace and determinant of a linear operator acting on the same space. For example, they can be defined as the trace and determinant of the matrix representation of $B^{-1} G$ with respect to an arbitrary chosen basis in $\mathbb {E}$ (the result is independent of the particular choice of basis). Alternatively, ${{\,\mathrm{tr}\,}}G$ and $\det G$ can be equivalently defined as the sum and product, respectively, of the eigenvalues of *G* with respect to *B*.

For a point $x \in \mathbb {E}$ and a real $r > 0$, by

$$\begin{aligned} {B}\left( {x, r} \right) :=\lbrace {y \in \mathbb {E} : \Vert x \Vert \le r} \rbrace , \end{aligned}$$

we denote the closed Euclidean ball with center *x* and radius *r*.

Given two solids^2^$Q, Q_0 \subseteq \mathbb {E}$, we can define the *relative volume* of *Q* with respect to $Q_0$ by ${{\,\mathrm{vol}\,}}(Q / Q_0) :={{\,\mathrm{vol}\,}}Q^e / {{\,\mathrm{vol}\,}}Q_0^e$, where *e* is an arbitrary basis in $\mathbb {E}$, $Q^e, Q_0^e \subseteq \mathbb {R}^n$ are the coordinate representations of the sets $Q, Q_0$ in the basis *e* and ${{\,\mathrm{vol}\,}}$ is the Lebesgue measure in $\mathbb {R}^n$. Note that the relative volume is independent of the particular choice of the basis *e*. Indeed, for any other basis *f*, we have $Q^e = T_f^e Q^f$, $Q_0^e = T_f^e Q_0^f$, where $T_f^e$ is the $n \times n$ change-of-basis matrix, so ${{\,\mathrm{vol}\,}}Q^e = (\det T_f^e) ({{\,\mathrm{vol}\,}}Q^f)$, ${{\,\mathrm{vol}\,}}Q_0^e = (\det T_f^e) ({{\,\mathrm{vol}\,}}Q_0^f)$ and hence ${{\,\mathrm{vol}\,}}Q^e / {{\,\mathrm{vol}\,}}Q_0^e = {{\,\mathrm{vol}\,}}Q^f / {{\,\mathrm{vol}\,}}Q_0^f$.

For us, it will be convenient to define the *volume* of a solid $Q \subseteq \mathbb {E}$ as the relative volume of *Q* with respect to the unit ball:

$$\begin{aligned} {{\,\mathrm{vol}\,}}Q :={{\,\mathrm{vol}\,}}(Q / {B}\left( {0, 1} \right) ). \end{aligned}$$

For an ellipsoid $W :=\lbrace {x \in \mathbb {E} : \langle {G x, x} \rangle \le 1} \rbrace$, where $G :\mathbb {E} \rightarrow \mathbb {E}^*$ is a self-adjoint positive definite linear operator, we have ${{\,\mathrm{vol}\,}}W = (\det G)^{-1/2}$.

## Convex problems and accuracy certificates

### Description and examples

In this paper, we consider numerical algorithms for solving *problems with convex structure*. The main examples of such problems are convex minimization problems, convex-concave saddle-point problems, convex Nash equilibrium problems, and variational inequalities with monotone operators.

The general formulation of a problem with convex structure involves two objects:

- Solid $Q \subseteq \mathbb {E}$ (called the *feasible set*), represented by the *Separation Oracle*: given any point $x \in \mathbb {E}$, this oracle can check whether $x \in {{\,\mathrm{int}\,}}Q$, and if not, it reports a vector $g_Q(x) \in \mathbb {E}^*\setminus \lbrace 0\rbrace$ which separates *x* from *Q*:
  4 $$\begin{aligned} \langle {g_Q(x), x - y} \rangle \ge 0, \quad \forall y \in Q. \end{aligned}$$
- Vector field $g :{{\,\mathrm{int}\,}}Q \rightarrow \mathbb {E}^*$, represented by the *First-Order Oracle*: given any point $x \in {{\,\mathrm{int}\,}}Q$, this oracle returns the vector *g*(*x*).

In what follows, we only consider the problems satisfying the following condition:

5 $$\begin{aligned} \exists x^* \in Q :\quad \langle {g(x), x - x^*} \rangle \ge 0, \quad \forall x \in {{\,\mathrm{int}\,}}Q. \end{aligned}$$

#### Remark 1

A careful reader may note that the notation $x^*$ overlaps with our general notation for the linear operator generated by a point *x* (see Sect. 1). However, there should be no risk of confusion since the precise meaning of $x^*$ can usually be easily inferred from the context.

A numerical algorithm for solving a problem with convex structure starts at some point $x_0 \in \mathbb {E}$. At each step $k \ge 0$, it queries the oracles at the current *test point* $x_k$ to obtain the new information about the problem, and then somehow uses this new information to form the next test point $x_{k+1}$. Depending on whether $x_k \in {{\,\mathrm{int}\,}}Q$, the *k*th step of the algorithm is called *productive* or *nonproductive*.

The total information, obtained by the algorithm from the oracles after $k \ge 1$ steps, comprises its *execution protocol* which consists of:

- The test points $x_0, \ldots , x_{k-1} \in \mathbb {E}$.
- The set of productive steps $I_k :=\lbrace {0 \le i \le k-1 : x_i \in {{\,\mathrm{int}\,}}Q} \rbrace$.
- The vectors $g_0, \ldots , g_{k-1} \in \mathbb {E}^*$ reported by the oracles: $g_i :=g(x_i)$, if $i \in I_k$, and $g_i :=g_Q(x_i)$, if $i \notin I_k$, $0 \le i \le k-1$.

An *accuracy certificate*, associated with the above execution protocol, is a nonnegative vector $\lambda :=(\lambda _0, \ldots , \lambda _{k-1})$ such that $S_k(\lambda ) :=\sum _{i \in I_k} \lambda _i > 0$ (and, in particular, $I_k \ne \emptyset$). Given any solid $\varOmega$, containing *Q*, we can define the following *residual* of $\lambda$ on $\varOmega$:

6 $$\begin{aligned} \epsilon _k(\lambda ) :=\max _{x \in \varOmega } \frac{1}{S_k(\lambda )} \sum _{i=0}^{k-1} \lambda _i \langle {g_i, x_i - x} \rangle , \end{aligned}$$

which is easily computable whenever $\varOmega$ is a simple set (e.g., a Euclidean ball). Note that

7 $$\begin{aligned} \epsilon _k(\lambda ) \ge \max _{x \in Q} \frac{1}{S_k(\lambda )} \sum _{i=0}^{k-1} \lambda _i \langle {g_i, x_i - x} \rangle \ge \max _{x \in Q} \frac{1}{S_k(\lambda )} \sum _{i \in I_k} \lambda _i \langle {g_i, x_i - x} \rangle \end{aligned}$$

and, in particular, $\epsilon _k(\lambda ) \ge 0$ in view of (5).

In what follows, we will be interested in the algorithms, which can produce accuracy certificates $\lambda ^{(k)}$ with $\epsilon _k(\lambda ^{(k)}) \rightarrow 0$ at a certain rate. This is a meaningful goal because, for all known instances of problems with convex structure, the residual $\epsilon _k(\lambda )$ upper bounds a certain natural inaccuracy measure for the corresponding problem. Let us briefly review some standard examples (for more examples, see [16, 18] and the references therein).

#### Example 1

**(Convex minimization problem)** Consider the problem

8 $$\begin{aligned} f^* :=\min _{x \in Q} f(x), \end{aligned}$$

where $Q \subseteq \mathbb {E}$ is a solid and $f :\mathbb {E} \rightarrow \mathbb {R} \cup \lbrace +\infty \rbrace$ is closed convex and finite on ${{\,\mathrm{int}\,}}Q$.

The First-Order Oracle for (8) is $g(x) :=f'(x)$, $x \in {{\,\mathrm{int}\,}}Q$, where $f'(x)$ is an arbitrary subgradient of *f* at *x*. Clearly, (5) holds for $x^*$ being any solution of (8).

One can verify that, in this example, the residual $\epsilon _k(\lambda )$ upper bounds the functional residual: for $\hat{x}_k :=\frac{1}{S_k(\lambda )} \sum _{i \in I_k} \lambda _i x_i$ or $x_k^* :=\mathop {\mathrm{argmin}}\limits \lbrace {f(x) : x \in X_k} \rbrace$, where $X_k :=\lbrace {x_i : i \in I_k} \rbrace$, we have $f(\hat{x}_k) - f^* \le \epsilon _k(\lambda )$ and $f(x_k^*) - f^* \le \epsilon _k(\lambda )$.

Moreover, $\epsilon _k(\lambda )$, in fact, upper bounds the primal-dual gap for a certain dual problem for (8). Indeed, let $f_* :\mathbb {E}^* \rightarrow \mathbb {R} \cup \lbrace +\infty \rbrace$ be the conjugate function of *f*. Then, we can represent (8) in the following dual form:

9 $$\begin{aligned} f^* = \min _{x \in Q} \max _{s \in {{\,\mathrm{dom}\,}}f_*} [\langle {s, x} \rangle - f_*(s)] = \max _{s \in {{\,\mathrm{dom}\,}}f_*} [-f_*(s) - \xi _Q(-s)], \end{aligned}$$

where ${{\,\mathrm{dom}\,}}f_* :=\lbrace {s \in \mathbb {E}^* : f_*(s) < +\infty } \rbrace$ and $\xi _Q(-s) :=\max _{x \in Q} \langle {-s, x} \rangle$. Denote $s_k :=\frac{1}{S_k(\lambda )} \sum _{i \in I_k} \lambda _i g_i$. Then, using (7) and the convexity of *f* and $f_*$, we obtain

$$\begin{aligned} \begin{aligned} \epsilon _k(\lambda )&\ge \frac{1}{S_k(\lambda )} \sum _{i \in I_k} \lambda _i \langle {g_i, x_i} \rangle + \xi _Q(-s_k) \\&= \frac{1}{S_k(\lambda )} \sum _{i \in I_k} \lambda _i [f(x_i) + f_*(g_i)] + \xi _Q(-s_k) \\&\ge f(\hat{x}_k) + f_*(s_k) + \xi _Q(-s_k). \end{aligned} \end{aligned}$$

Thus, $\hat{x}_k$ and $s_k$ are $\epsilon _k(\lambda )$-approximate solutions (in terms of function value) to problems (8) and (9), respectively. Note that the same is true if we replace $\hat{x}_k$ with $x_k^*$.

#### Example 2

**(Convex-concave saddle-point problem)** Consider the following problem: Find $(u^*, v^*) \in U \times V$ such that

10 $$\begin{aligned} f(u^*, v) \le f(u^*, v^*) \le f(u, v^*), \quad \forall (u, v) \in U \times V, \end{aligned}$$

where *U*, *V* are solids in some finite-dimensional vector spaces $\mathbb {E}_u$, $\mathbb {E}_v$, respectively, and $f :U \times V \rightarrow \mathbb {R}$ is a continuous function which is *convex-concave*, i.e., $f(\cdot , v)$ is convex and $f(u, \cdot )$ is concave for any $u \in U$ and any $v \in V$.

In this example, we set $\mathbb {E} :=\mathbb {E}_u \times \mathbb {E}_v$, $Q :=U \times V$ and use the First-Order Oracle

$$\begin{aligned} g(x) :=(f'_u(x), -f'_v(x)), \quad x :=(u, v) \in {{\,\mathrm{int}\,}}Q, \end{aligned}$$

where $f'_u(x)$ is an arbitrary subgradient of $f(\cdot , v)$ at *u* and $f'_v(y)$ is an arbitrary supergradient of $f(u, \cdot )$ at *v*. Then, for any $x :=(u, v) \in {{\,\mathrm{int}\,}}Q$ and any $x' :=(u', v') \in Q$,

11 $$\begin{aligned} \langle {g(x), x - x'} \rangle = \langle {f'_u(x), u - u'} \rangle - \langle {f'_v(x), v - v'} \rangle \ge f(u, v') - f(u', v). \end{aligned}$$

In particular, (5) holds for $x^* :=(u^*, v^*)$ in view of (10).

Let $\phi :U \rightarrow \mathbb {R}$ and $\psi :V \rightarrow \mathbb {R}$ be the functions

$$\begin{aligned} \phi (u) :=\max _{v \in V} f(u, v), \qquad \psi (v) :=\min _{u \in U} f(u, v). \end{aligned}$$

In view of (10), we have $\psi (v) \le f(u^*, v^*) \le \phi (u)$ for all $(u, v) \in U \times V$. Therefore, the difference $\phi (u) - \psi (v)$ (called the *primal-dual gap*) can be used for measuring the quality of an approximate solution $x :=(u, v) \in Q$ to problem (10).

Denoting $\hat{x}_k :=\frac{1}{S_k(\lambda )} \sum _{i \in I_k} \lambda _i x_i =:(\hat{u}_k, \hat{v}_k)$ and using (7), we obtain

$$\begin{aligned} \begin{aligned} \epsilon _k(\lambda )&\ge \max _{x \in Q} \frac{1}{S_k(\lambda )} \sum _{i \in I_k} \lambda _i \langle {g_i, x_i - x} \rangle \\&\ge \max _{u \in U, v \in V} \frac{1}{S_k(\lambda )} \sum _{i \in I_k}\lambda _i [f(u_i, v) - f(u, v_i)] \\&\ge \max _{u \in U, v \in V} [f(\hat{u}_k, v) - f(u, \hat{v}_k)] = \phi (\hat{u}_k) - \psi (\hat{v}_k), \end{aligned} \end{aligned}$$

where the second inequality is due to (11) and the last one follows from the convexity-concavity of *f*. Thus, the residual $\epsilon _k(\lambda )$ upper bounds the primal-dual gap for the approximate solution $\hat{x}_k$.

#### Example 3

**(Variational inequality with monotone operator)** Let $Q \subseteq \mathbb {E}$ be a solid and let $V :Q \rightarrow \mathbb {E}^*$ be a continuous operator which is *monotone*, i.e., $\langle {V(x) - V(y), x - y} \rangle \ge 0$ for all $x, y \in Q$. The goal is to solve the following (weak) *variational inequality*:

12 $$\begin{aligned} \text {Find}\, x^* \in Q :\quad \langle {V(x), x - x^*} \rangle \ge 0, \quad \forall x \in Q. \end{aligned}$$

Since *V* is continuous, this problem is equivalent to its strong variant: find $x^* \in Q$ such that $\langle {V(x^*), x - x^*} \rangle \ge 0$ for all $x \in Q$.

A standard tool for measuring the quality of an approximate solution to (12) is the *dual gap function*, introduced in [1]:

$$\begin{aligned} f(x) :=\max _{y \in Q} \langle {V(y), x - y} \rangle , \qquad x \in Q. \end{aligned}$$

It is easy to see that *f* is a convex nonnegative function which equals 0 exactly at the solutions of (12).

In this example, the First-Order Oracle is defined by $g(x) :=V(x)$ for any $x \in {{\,\mathrm{int}\,}}Q$. Denote $\hat{x}_k :=\frac{1}{S_k(\lambda )} \sum _{i \in I_k} \lambda _i x_i$. Then, using (7) and the monotonicity of *V*, we obtain

$$\begin{aligned} \begin{aligned} \epsilon _k(\lambda )&\ge \max _{x \in Q} \frac{1}{S_k(\lambda )} \sum _{i \in I_k} \lambda _i \langle {V(x_i), x_i - x} \rangle \\&\ge \max _{x \in Q} \frac{1}{S_k(\lambda )} \sum _{i \in I_k} \lambda _i \langle {V(x), x_i - x} \rangle = f(\hat{x}_k). \end{aligned} \end{aligned}$$

Thus, $\epsilon _k(\lambda )$ upper bounds the dual gap function for the approximate solution $\hat{x}_k$.

### Establishing convergence of residual

For the algorithms, considered in this paper, instead of accuracy certificates and residuals, it turns out to be more convenient to speak about closely related notions of *accuracy semicertificates* and *gaps*, which we now introduce.

As before, let $x_0, \dots , x_{k-1}$ be the test points, generated by the algorithm after $k \ge 1$ steps, and let $g_0, \dots , g_{k-1}$ be the corresponding oracle outputs. An *accuracy semicertificate*, associated with this information, is a nonnegative vector $\lambda :=(\lambda _0, \dots , \lambda _{k-1})$ such that $\varGamma _k(\lambda ) :=\sum _{i=0}^{k-1} \lambda _i {\Vert {g_i} \Vert }_* > 0$. Given any solid $\varOmega$, containing *Q*, the *gap* of $\lambda$ on $\varOmega$ is defined in the following way:

13 $$\begin{aligned} \delta _k(\lambda ) :=\max _{x \in \varOmega } \frac{1}{\varGamma _k(\lambda )} \sum _{i=0}^{k-1} \lambda _i \langle {g_i, x_i - x} \rangle . \end{aligned}$$

Comparing these definitions with those of accuracy certificate and residual, we see that the only difference between them is that now we use a different “normalizing” coefficient: $\varGamma _k(\lambda )$ instead of $S_k(\lambda )$. Also, in the definitions of semicertificate and gap, we do not make any distinction between productive and nonproductive steps. Note that $\delta _k(\lambda ) \ge 0$.

Let us demonstrate that by making the gap sufficiently small, we can make the corresponding residual sufficiently small as well. For this, we need the following standard assumption about our problem with convex structure (see, e.g., [16]).

#### Assumption 1

The vector field *g*, reported by the First-Order Oracle, is semibounded:

$$\begin{aligned} \langle {g(x), y - x} \rangle \le V, \quad \forall x \in {{\,\mathrm{int}\,}}Q, \ \forall y \in Q. \end{aligned}$$

A classical example of a semibounded field is a bounded one: if there is $M \ge 0$, such that ${\Vert {g(x)} \Vert }_* \le M$ for all $x \in {{\,\mathrm{int}\,}}Q$, then *g* is semibounded with $V :=M D$, where *D* is the diameter of *Q*. However, there exist other examples. For instance, if *g* is the subgradient field of a convex function $f :\mathbb {E} \rightarrow \mathbb {R} \cup \lbrace +\infty \rbrace$, which is finite and continuous on *Q*, then *g* is semibounded with $V :=\max _Q f - \min _Q f$ (variation of *f* on *Q*); however, *g* is not bounded if *f* is not Lipschitz continuous (e.g., $f(x) :=-\sqrt{x}$ on $Q :=[{0, 1} ]$). Another interesting example is the subgradient field *g* of a $\nu$-self-concordant barrier $f :\mathbb {E} \rightarrow \mathbb {R} \cup \lbrace +\infty \rbrace$ for the set *Q*; in this case, *g* is semibounded with $V :=\nu$ (see, e.g., [19, Theorem 5.3.7]), while $f(x) \rightarrow +\infty$ at the boundary of *Q*.

#### Lemma 1

Let $\lambda$ be a semicertificate such that $\delta _k(\lambda ) < r$, where *r* is the largest of the radii of Euclidean balls contained in *Q*. Then, $\lambda$ is a certificate and

$$\begin{aligned} \epsilon _k(\lambda ) \le \frac{\delta _k(\lambda )}{r - \delta _k(\lambda )} V. \end{aligned}$$

#### Proof

Denote $\delta _k :=\delta _k(\lambda )$, $\varGamma _k :=\varGamma _k(\lambda )$, $S_k :=S_k(\lambda )$. Let $\bar{x} \in Q$ be such that ${B}\left( {\bar{x}, r} \right) \subseteq Q$. For each $0 \le i \le k-1$, let $z_i$ be a maximizer of $z \mapsto \langle {g_i, z - \bar{x}} \rangle$ on ${B}\left( {\bar{x}, r} \right)$. Then, for any $0 \le i \le k-1$, we have $\langle {g_i, \bar{x} - x_i} \rangle = \langle {g_i, z_i - x_i} \rangle - r {\Vert {g_i} \Vert }_*$ with $z_i \in Q$. Therefore,

14 $$\begin{aligned} \sum _{i=0}^{k-1} \lambda _i \langle {g_i, \bar{x} - x_i} \rangle = \sum _{i=0}^{k-1} \lambda _i \langle {g_i, z_i - x_i} \rangle - r \varGamma _k \le S_k V - r \varGamma _k, \end{aligned}$$

where the inequality follows from the separation property (4) and Assumption 1.

Let $x \in \varOmega$ be arbitrary. Denoting $y :=\bigl ( \delta _k \bar{x} + (r - \delta _k) x \bigr ) / r \in \varOmega$, we obtain

15 $$\begin{aligned} (r - \delta _k) \sum _{i=0}^{k-1} \lambda _i \langle {g_i, x_i - x} \rangle&= r \sum _{i=0}^{k-1} \lambda _i \langle {g_i, x_i - y} \rangle + \delta _k \sum _{i=0}^{k-1} \lambda _i \langle {g_i, \bar{x} - x_i} \rangle \nonumber \\&\le r \delta _k \varGamma _k + \delta _k \sum _{i=0}^{k-1} \lambda _i \langle {g_i, \bar{x} - x_i} \rangle \le \delta _k S_k V, \end{aligned}$$

where the inequalities follow from the definition (13) of $\delta _k$ and (14), respectively.

It remains to show that $\lambda$ is a certificate, i.e., $S_k > 0$. But this is simple. Indeed, if $S_k = 0$, then, taking $x :=\bar{x}$ in (15) and using (14), we get $0 \ge \sum _{i=0}^{k-1} \lambda _i \langle {g_i, x_i - \bar{x}} \rangle \ge r \varGamma _k$, which contradicts our assumption that $\lambda$ is a semicertificate, i.e., $\varGamma _k > 0$. $\square$

According to Lemma 1, from the convergence rate of the gap $\delta _k(\lambda ^{(k)})$ to zero, we can easily obtain the corresponding convergence rate of the residual $\epsilon _k(\lambda ^{(k)})$. In particular, to ensure that $\epsilon _k(\lambda ^{(k)}) \le \epsilon$ for some $\epsilon > 0$, it suffices to make $\delta _k(\lambda ^{(k)}) \le \delta (\epsilon ) :=\epsilon r / (\epsilon + V)$. For this reason, in the rest of this paper, we can focus our attention on studying the convergence rate only for the gap.

## General algorithmic scheme

Consider the general scheme presented in Algorithm 1. This scheme works with an arbitrary oracle $\mathcal {G} :\mathbb {E} \rightarrow \mathbb {E}^*$ satisfying the following condition:

16 $$\begin{aligned} \exists x^* \in {B}\left( {x_0, R} \right) :\quad \langle {\mathcal {G}(x), x - x^*} \rangle \ge 0, \quad \forall x \in \mathbb {E}. \end{aligned}$$

The point $x^*$ from (16) is typically called a *solution* of our problem. For the general problem with convex structure, represented by the First-Order Oracle *g* and the Separation Oracle $g_Q$ for the solid *Q*, the oracle $\mathcal {G}$ is usually defined as follows: $\mathcal {G}(x) :=g(x)$, if $x \in {{\,\mathrm{int}\,}}Q$, and $\mathcal {G}(x) :=g_Q(x)$, otherwise. To ensure that (16) holds, the constant *R* needs to be chosen sufficiently big so that $Q \subseteq {B}\left( {x_0, R} \right)$.
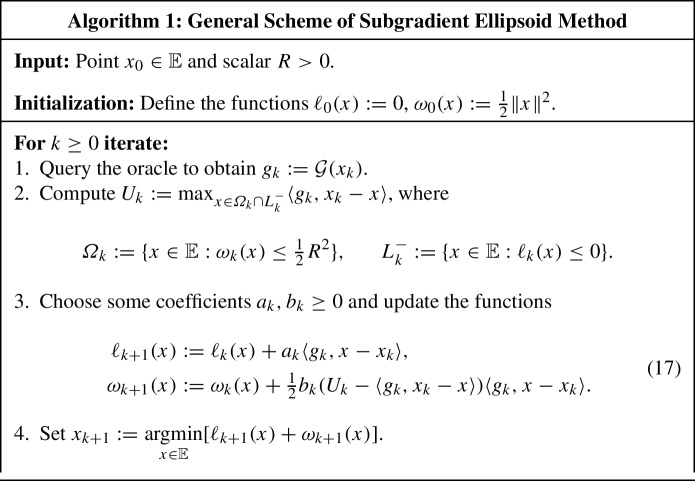


Note that, in Algorithm 1, $\omega _k$ are strictly convex quadratic functions and $\ell _k$ are affine functions. Therefore, the sets $\varOmega _k$ are certain ellipsoids and $L_k^-$ are certain halfspaces (possibly degenerate).

Let us show that Algorithm 1 is a cutting-plane scheme in which the sets $\varOmega _k \cap L_k^-$ are the localizers of the solution $x^*$.

### Lemma 2

In Algorithm 1, for all $k \ge 0$, we have $x^* \in \varOmega _k \cap L_k^-$ and $\hat{Q}_{k+1} \subseteq \varOmega _{k+1} \cap L_{k+1}^-$, where $\hat{Q}_{k+1} :=\lbrace {x \in \varOmega _k \cap L_k^- : \langle {g_k, x - x_k} \rangle \le 0} \rbrace$.

### Proof

Let us prove the claim by induction. Clearly, $\varOmega _0 = {B}\left( {x_0, R} \right)$, $L_0^- = \mathbb {E}$, hence $\varOmega _0 \cap L_0^- = {B}\left( {x_0, R} \right) \ni x^*$ by (16). Suppose we have already proved that $x^* \in \varOmega _k \cap L_k^-$ for some $k \ge 0$. Combining this with (16), we obtain $x^* \in \hat{Q}_{k+1}$, so it remains to show that $\hat{Q}_{k+1} \subseteq \varOmega _{k+1} \cap L_{k+1}^-$. Let $x \in \hat{Q}_{k+1}$ ($\subseteq \varOmega _k \cap L_k^-$) be arbitrary. Note that $0 \le \langle {g_k, x_k - x} \rangle \le U_k$. Hence, by (17), $\ell _{k+1}(x) \le \ell _k(x) \le 0$ and $\omega _{k+1}(x) \le \omega _k(x) \le \frac{1}{2} R^2$, which means that $x \in \varOmega _{k+1} \cap L_{k+1}^-$. $\square$

Next, let us establish an important representation of the ellipsoids $\varOmega _k$ via the functions $\ell _k$ and the test points $x_k$. For this, let us define $G_k :=\nabla ^2\omega _k(0)$ for each $k \ge 0$. Observe that these operators satisfy the following simple relations (cf. (17)):

18 $$\begin{aligned} G_0 = B, \qquad G_{k+1} = G_k + b_k g_k g_k^*, \quad k \ge 0. \end{aligned}$$

Also, let us define the sequence $R_k > 0$ by the recurrence

19 $$\begin{aligned} R_0 = R, \qquad R_{k+1}^2 = R_k^2 + ( a_k + \tfrac{1}{2} b_k U_k )^2 \frac{\Vert {g_k}\Vert _{G_k}^2}{1 + b_k \Vert {g_k}\Vert _{G_k}^{2}}, \quad k \ge 0. \end{aligned}$$

### Lemma 3

In Algorithm 1, for all $k \ge 0$, we have

$$\begin{aligned} \varOmega _k = \lbrace {x \in \mathbb {E} : -\ell _k(x) + \tfrac{1}{2} {\Vert x - x_k \Vert }_{G_k}^2 \le \tfrac{1}{2} R_k^2 } \rbrace . \end{aligned}$$

In particular, for all $k \ge 0$ and all $x \in \varOmega _k \cap L_k^-$, we have ${\Vert x - x_k \Vert }_{G_k} \le R_k$.

### Proof

Let $\psi _k :\mathbb {E} \rightarrow \mathbb {R}$ be the function $\psi _k(x) :=\ell _k(x) + \omega _k(x)$. Note that $\psi _k$ is a quadratic function with Hessian $G_k$ and minimizer $x_k$. Hence, for any $x \in \mathbb {E}$, we have

20 $$\begin{aligned} \psi _k(x) = \psi _k^* + \tfrac{1}{2} {\Vert x - x_k \Vert }_{G_k}^2, \end{aligned}$$

where $$\psi _k^* :=\min _{x \in \mathbb {E}} \psi _k(x)$$.

Let us compute $\psi _k^*$. Combining (17), (18) and (20), for any $x \in \mathbb {E}$, we obtain

21 $$\begin{aligned} \psi _{k+1}(x)&= \psi _k(x) + (a_k + \tfrac{1}{2} b_k U_k) \langle {g_k, x - x_k} \rangle + \tfrac{1}{2} b_k \langle {g_k, x - x_k} \rangle ^2\nonumber \\&= \psi _k^* + \tfrac{1}{2} {\Vert x - x_k \Vert }_{G_k}^2 + (a_k + \tfrac{1}{2} b_k U_k) \langle {g_k, x - x_k} \rangle + \tfrac{1}{2} b_k \langle {g_k, x - x_k } \rangle ^2\nonumber \\&= \psi _k^* + \tfrac{1}{2} {\Vert x - x_k \Vert }_{G_{k+1}}^2 + (a_k + \tfrac{1}{2} b_k U_k) \langle {g_k, x - x_k} \rangle , \end{aligned}$$

Therefore,

22 $$\begin{aligned} \begin{aligned} \psi _{k+1}^*&= \psi _k^* - \tfrac{1}{2} ( a_k + \tfrac{1}{2} b_k U_k )^2 {\Vert {g_k}\Vert }_{G_{k+1}}^2 \\&= \psi _k^* - \tfrac{1}{2} ( a_k + \tfrac{1}{2} b_k U_k )^2 \frac{{\Vert {g_k}\Vert }_{G_k}^2}{ 1 + b_k {\Vert {g_k}\Vert }_{G_k}^2}, \end{aligned} \end{aligned}$$

where the last identity follows from the fact that $G_{k+1}^{-1} g_k = G_k^{-1} g_k / (1 + b_k {\Vert {g_k}\Vert }_{G_k}^2)$ (since $G_{k+1} G_k^{-1} g_k = (1 + b_k {\Vert {g_k}\Vert }_{G_k}^2) g_k$ in view of (18)). Since (22) is true for any $k \ge 0$ and since $\psi _0^* = 0$, we thus obtain, in view of (19),

23 $$\begin{aligned} \psi _k^* = \tfrac{1}{2} (R^2 - R_k^2). \end{aligned}$$

Let $x \in \varOmega _k$ be arbitrary. Using the definition of $\psi _k(x)$ and (23), we obtain

$$\begin{aligned} -\ell _k(x) + \tfrac{1}{2} {\Vert x - x_k \Vert }_{G_k}^2 = \omega _k(x) - \psi _k^* = \omega _k(x) + \tfrac{1}{2} (R_k^2 - R^2). \end{aligned}$$

Thus, $x \in \varOmega _k \iff \omega _k(x) \le \frac{1}{2} R^2 \iff -\ell _k(x) + \frac{1}{2} {\Vert x - x_k \Vert }_{G_k}^2 \le \frac{1}{2} R_k^2$. In particular, for any $x \in \varOmega _k \cap L_k^-$, we have $\ell _k(x) \le 0$ and ${\Vert x - x_k \Vert }_{G_k} \le R_k$. $\square$

Lemma 3 has several consequences. First, we see that the localizers $\varOmega _k \cap L_k^-$ are contained in the ellipsoids $\lbrace {x : {\Vert x - x_k \Vert }_{G_k} \le R_k} \rbrace$ whose centers are the test points $x_k$.

Second, we get a uniform upper bound on the function $-\ell _k$ on the ellipsoid $\varOmega _k$: $-\ell _k(x) \le \frac{1}{2} R_k^2$ for all $x \in \varOmega _k$. This observation leads us to the following definition of the *sliding gap*:

24 $$\begin{aligned} \varDelta _k :=\max _{x \in \varOmega _k} \frac{1}{\varGamma _k} [-\ell _k(x)] = \max _{x \in \varOmega _k} \frac{1}{\varGamma _k} \sum _{i=0}^{k-1} a_i \langle {g_i, x_i - x} \rangle , \quad k \ge 1, \end{aligned}$$

provided that $\varGamma _k :=\sum _{i=0}^{k-1} a_i {\Vert {g_i} \Vert }_* > 0$. According to our observation, we have

25 $$\begin{aligned} \varDelta _k \le \frac{R_k^2}{2 \varGamma _k}. \end{aligned}$$

At the same time, $\varDelta _k \ge 0$ in view of Lemma 2 and 16

Comparing the definition (24) of the sliding gap $\varDelta _k$ with the definition (13) of the gap $\delta _k(a^{(k)})$ for the semicertificate $a^{(k)} :=(a_0, \dots , a_{k-1})$, we see that they are almost identical. The only difference between them is that the solid $\varOmega _k$, over which the maximum is taken in the definition of the sliding gap, depends on the iteration counter *k*. This seems to be unfortunate because we cannot guarantee that *each* $\varOmega _k$ contains the feasible set *Q* (as required in the definition of gap) even if so does the initial solid $\varOmega _0 = {B}\left( {x_0, R} \right)$. However, this problem can be dealt with. Namely, in Sect. 5, we will show that the semicertificate $a^{(k)}$ can be efficiently converted into another semicertificate $\lambda ^{(k)}$ for which $\delta _k(\lambda ^{(k)}) \le \varDelta _k$ when taken over the initial solid $\varOmega :=\varOmega _0$. Thus, the sliding gap $\varDelta _k$ is a meaningful measure of convergence rate of Algorithm 1, and it makes sense to call the coefficients $a^{(k)}$ a *preliminary semicertificate*.

Let us now demonstrate that, for a suitable choice of the coefficients $a_k$ and $b_k$ in Algorithm 1, we can ensure that the sliding gap $\varDelta _k$ converges to zero.

### Remark 2

From now on, in order to avoid taking into account some trivial degenerate cases, it will be convenient to make the following minor technical assumption:

$$\begin{aligned} \text {In Algorithm}\, {1}, g_k \ne 0\,\text { for all}\, k \ge 0. \end{aligned}$$

Indeed, when the oracle reports $g_k = 0$ for some $k \ge 0$, it usually means that the test point $x_k$, at which the oracle was queried, is, in fact, an exact solution to our problem. For example, if the standard oracle for a problem with convex structure has reported $g_k = 0$, we can terminate the method and return the certificate $\lambda :=(0, \dots , 0, 1)$ for which the residual $\epsilon _k(\lambda ) = 0$.

Let us choose the coefficients $a_k$ and $b_k$ in the following way:

26 $$\begin{aligned} a_k :=\frac{\alpha _k R + \frac{1}{2} \theta \gamma R_k}{ {\Vert g_k \Vert }_{G_k}^*}, \qquad b_k :=\frac{\gamma }{{\Vert {g_k}\Vert }_{G_k}^2}, \qquad k \ge 0, \end{aligned}$$

where $\alpha _k, \theta , \gamma \ge 0$ are certain coefficients to be chosen later.

According to (25), to estimate the convergence rate of the sliding gap, we need to estimate the rate of growth of the coefficients $R_k$ and $\varGamma _k$ from above and below, respectively. Let us do this.

### Lemma 4

In Algorithm 1 with parameters (26), for all $k \ge 0$, we have

27 $$\begin{aligned} R_k^2 \le [q_c(\gamma )]^k C_k R^2, \end{aligned}$$

where $q_c(\gamma ) :=1 + \frac{c \gamma ^2}{2 (1 + \gamma )}$, $c :=\tfrac{1}{2} (\tau + 1) (\theta + 1)^2$, $C_k :=1 + \frac{\tau + 1}{\tau } \sum _{i=0}^{k-1} \alpha _i^2$ and $\tau > 0$ can be chosen arbitrarily. Moreover, if $\alpha _k = 0$ for all $k \ge 0$, then, $R_k^2 = [q_c(\gamma )]^k R^2$ for all $k \ge 0$ with $c :=\frac{1}{2} (\theta + 1)^2$.

### Proof

By the definition of $U_k$ and Lemma 3, we have

28 $$\begin{aligned} U_k = \max _{x \in \varOmega _k \cap L_k^-} \langle {g_k, x_k - x} \rangle \le \max _{{\Vert x - x_k \Vert }_{G_k} \le R_k} \langle {g_k, x_k - x} \rangle = R_k {\Vert g_k \Vert }_{G_k}^*. \end{aligned}$$

At the same time, $U_k \ge 0$ in view of Lemma 2 and (16). Hence,

$$\begin{aligned} \begin{aligned} (a_k + \tfrac{1}{2} b_k U_k)^2 \frac{{\Vert {g_k}\Vert }_{G_k}^2}{ 1 + b_k {\Vert {g_k}\Vert }_{G_k}^2}&\le (a_k + \tfrac{1}{2} b_k R_k {\Vert g_k \Vert }_{G_k}^*)^2 \frac{{\Vert {g_k}\Vert }_{G_k}^2}{ 1 + b_k {\Vert {g_k}\Vert }_{G_k}^2} \\&= \frac{1}{1 + \gamma } \bigl ( \alpha _k R + \tfrac{1}{2} (\theta + 1) \gamma R_k \bigr )^2, \end{aligned} \end{aligned}$$

where the identity follows from (26). Combining this with (19), we obtain

29 $$\begin{aligned} R_{k+1}^2 \le R_k^2 + \frac{1}{1 + \gamma } \bigl ( \alpha _k R + \tfrac{1}{2} (\theta + 1) \gamma R_k \bigr )^2. \end{aligned}$$

Note that, for any $\xi _1, \xi _2 \ge 0$ and any $\tau > 0$, we have

$$\begin{aligned} (\xi _1 + \xi _2)^2 = \xi _1^2 + 2 \xi _1 \xi _2 + \xi _2^2 \le \frac{\tau + 1}{\tau } \xi _1^2 + (\tau + 1) \xi _2^2 = (\tau + 1) \Bigl ( \frac{1}{\tau } \xi _1^2 + \xi _2^2 \Bigr ) \end{aligned}$$

(look at the minimum of the right-hand side in $\tau$). Therefore, for arbitrary $\tau > 0$,

$$\begin{aligned} R_{k+1}^2 \le R_k^2 + \frac{\tau + 1}{1 + \gamma } \Bigl ( \frac{1}{\tau } \alpha _k^2 R^2 + \tfrac{1}{4} (\theta + 1)^2 \gamma ^2 R_k^2 \Bigr ) = q R_k^2 + \beta _k R^2, \end{aligned}$$

where we denote $q :=q_c(\gamma ) \ge 1$ and $\beta _k :=\frac{\tau + 1}{\tau (1 + \gamma )} \alpha _k^2$. Dividing both sides by $q^{k+1}$, we get

$$\begin{aligned} \frac{R_{k+1}^2}{q^{k+1}} \le \frac{R_k^2}{q^k} + \frac{\beta _k R^2}{q^{k+1}}. \end{aligned}$$

Since this is true for any $k \ge 0$, we thus obtain, in view of (19), that

$$\begin{aligned} \frac{R_k^2}{q^k} \le \frac{R_0^2}{q^0} + R^2 \sum _{i=0}^{k-1} \frac{\beta _i}{q^{i+1}} = \biggl ( 1 + \sum _{i=0}^{k-1} \frac{\beta _i}{q^{i+1}} \biggr ) R^2, \end{aligned}$$

Multiplying both sides by $q^k$ and using that $\frac{\beta _i}{q^{i+1}} \le \frac{\tau + 1}{\tau } \alpha _i^2$, we come to (27).

When $\alpha _k = 0$ for all $k \ge 0$, we have $\ell _k = 0$ and $L_k^- = \mathbb {E}$ for all $k \ge 0$. Therefore, by Lemma 3, $\varOmega _k = \lbrace {x : {\Vert x - x_k \Vert }_{G_k} \le R_k} \rbrace$ and hence (28) is, in fact, an equality. Consequently, (29) becomes $R_{k+1}^2 = R_k^2 + \frac{c \gamma ^2}{2 (1 + \gamma )} R_k^2 = q_c(\gamma ) R_k^2$, where $c :=\frac{1}{2} (\theta + 1)^2$. $\square$

### Remark 3

From the proof, one can see that the quantity $C_k$ in Lemma 4 can be improved up to $C_k' :=1 + \frac{\tau + 1}{\tau (1 + \gamma )} \sum _{i=0}^{k-1} \frac{\alpha _i^2}{[q_c(\gamma )]^{i+1}}$.

### Lemma 5

In Algorithm 1 with parameters (26), for all $k \ge 1$, we have

30 $$\begin{aligned} \varGamma _k \ge R \Bigl ( \, \sum _{i=0}^{k-1} \alpha _i + \tfrac{1}{2} \theta \sqrt{\gamma n \bigl [ (1 + \gamma )^{k/n} - 1 \bigr ]} \, \Bigr ). \end{aligned}$$

### Proof

By the definition of $\varGamma _k$ and (26), we have

$$\begin{aligned} \varGamma _k = \sum _{i=0}^{k-1} a_i {\Vert {g_i} \Vert }_* = R \sum _{i=0}^{k-1} \alpha _i \rho _i + \tfrac{1}{2} \theta \gamma \sum _{i=0}^{k-1} R_i \rho _i, \end{aligned}$$

where $\rho _i :={\Vert {g_i} \Vert }_* / {\Vert g_i \Vert }_{G_i}^*$. Let us estimate each sum from below separately.

For the first sum, we can use the trivial bound $\rho _i \ge 1$, which is valid for any $i \ge 0$ (since $G_i \succeq B$ in view of (18)). This gives us $\sum _{i=0}^{k-1} \alpha _i \rho _i \ge \sum _{i=0}^{k-1} \alpha _i$.

Let us estimate the second sum. According to (19), for any $i \ge 0$, we have $R_i \ge R$. Hence, $\sum _{i=0}^{k-1} R_i \rho _i \ge R \sum _{i=0}^{k-1} \rho _i \ge R \left( \sum _{i=0}^{k-1} \rho _i^2 \right) ^{1/2}$ and it remains to lower bound $\sum _{i=0}^{k-1} \rho _i^2$. By 18 and 26, $G_0 = B$ and $G_{i+1} = G_i + \gamma g_i g_i^*/ \Vert {g_i}\Vert _{G_i}^2$ for all $i \ge 0$. Therefore,

$$\begin{aligned} \begin{aligned} \sum _{i=0}^{k-1} \rho _i^2&= \frac{1}{\gamma } \sum _{i=0}^{k-1} ( {{\,\mathrm{tr}\,}}G_{i+1} - {{\,\mathrm{tr}\,}}G_i ) = \frac{1}{\gamma } ({{\,\mathrm{tr}\,}}G_k - {{\,\mathrm{tr}\,}}B) = \frac{1}{\gamma } ({{\,\mathrm{tr}\,}}G_k - n) \\&\ge \frac{n}{\gamma } \bigl [ (\det G_k)^{1/n} - 1 \bigr ] = \frac{n}{\gamma } \bigl [ (1 + \gamma )^{k/n} - 1 \bigr ], \end{aligned} \end{aligned}$$

where we have applied the arithmetic-geometric mean inequality. Combining the obtained estimates, we get (30). $\square$

## Main instances of general scheme

Let us now consider several possibilities for choosing the coefficients $\alpha _k$, $\theta$ and $\gamma$ in (26).

### Subgradient method

The simplest possibility is to choose

$$\begin{aligned} \alpha _k > 0, \qquad \theta :=0, \qquad \gamma :=0. \end{aligned}$$

In this case, $b_k = 0$ for all $k \ge 0$, so $G_k = B$ and $\omega _k(x) = \omega _0(x) = \frac{1}{2} \Vert x \Vert ^2$ for all $x \in \mathbb {E}$ and all $k \ge 0$ (see (17) and (18)). Consequently, the new test points $x_{k+1}$ in Algorithm 1 are generated according to the following rule:

$$\begin{aligned} x_{k+1} = \mathop {\mathrm{argmin}}\limits _{x \in \mathbb {E}} \Bigl [ \, \sum _{i=0}^k a_i \langle {g_i, x - x_i} \rangle + \tfrac{1}{2} \Vert x \Vert ^2 \Bigr ], \end{aligned}$$

where $a_i = \alpha _i R / {\Vert {g_i} \Vert }_*$. Thus, Algorithm 1 is the Subgradient Method: $x_{k+1} = x_k - a_k g_k$.

In this example, each ellipsoid $\varOmega _k$ is simply a ball: $\varOmega _k = {B}\left( {x_0, R} \right)$ for all $k \ge 0$. Hence, the sliding gap $\varDelta _k$, defined in (24), does not “slide” and coincides with the gap of the semicertificate $a :=(a_0, \dots , a_{k-1})$ on the solid ${B}\left( {x_0, R} \right)$:

$$\begin{aligned} \varDelta _k = \max _{x \in {B}\left( {x_0, R} \right) } \frac{1}{\varGamma _k} \sum _{i=0}^{k-1} a_i \langle {g_i, x_i - x} \rangle . \end{aligned}$$

In view of Lemmas 4 and 5, for all $k \ge 1$, we have

$$\begin{aligned} R_k^2 \le \Bigl (1 + \sum _{i=0}^{k-1} \alpha _i^2 \Bigr ) R^2, \qquad \varGamma _k \ge R \sum _{i=0}^{k-1} \alpha _i \end{aligned}$$

(tend $\tau \rightarrow +\infty$ in Lemma 4). Substituting these estimates into (25), we obtain the following well-known estimate for the gap in the Subgradient Method:

$$\begin{aligned} \varDelta _k \le \frac{1 + \sum _{i=0}^{k-1} \alpha _i^2}{ 2 \sum _{i=0}^{k-1} \alpha _i} R. \end{aligned}$$

The standard strategies for choosing the coefficients $\alpha _i$ are as follows (see, e.g., sect. 3.2.3 in [19]):

1. We fix in advance the number of iterations $k \ge 1$ of the method and use *constant* coefficients $\alpha _i :=\frac{1}{\sqrt{k}}$, $0 \le i \le k - 1$. This corresponds to the so-called *Short-Step* Subgradient Method, for which we have
  $$\begin{aligned} \varDelta _k \le \frac{R}{\sqrt{k}}. \end{aligned}$$
2. Alternatively, we can use *time-varying* coefficients $\alpha _i :=\frac{1}{\sqrt{i + 1}}$, $i \ge 0$. This approach does not require us to fix in advance the number of iterations *k*. However, the corresponding convergence rate estimate becomes slightly worse:
  $$\begin{aligned} \varDelta _k \le \frac{\ln k + 2}{2 \sqrt{k}} R. \end{aligned}$$
  (Indeed, $$\sum _{i=0}^{k-1} \alpha _i^2 = \sum _{i=1}^k \frac{1}{i} \le \ln k + 1$$, while $$\sum _{i=0}^{k-1} \alpha _i \ge \sqrt{k}$$.)

#### Remark 4

If we allow projections onto the feasible set, then, for the resulting Subgradient Method with time-varying coefficients $\alpha _i$, one can establish the $O(1/\sqrt{k})$ convergence rate for the “truncated” gap

$$\begin{aligned} \varDelta _{k_0, k} :=\max _{x \in {B}\left( {x_0, R} \right) } \frac{1}{\varGamma _{k_0, k}} \sum _{i=k_0}^{k} a_i \langle {g_i, x_i - x} \rangle , \end{aligned}$$

where $\varGamma _{k_0, k} :=\sum _{i=k_0}^k a_i {\Vert {g_i} \Vert }_*$, $k_0 :=\lceil k/2 \rceil$. For more details, see sect. 5.2.1 in [2] or sect. 3.1.1 in [12].

### Standard ellipsoid method

Another extreme choice is the following one:

31 $$\begin{aligned} \alpha _k :=0, \qquad \theta :=0, \qquad \gamma > 0. \end{aligned}$$

For this choice, we have $a_k = 0$ for all $k \ge 0$. Hence, $\ell _k = 0$ and $L_k^- = \mathbb {E}$ for all $k \ge 0$. Therefore, the localizers in this method are the following ellipsoids (see Lemma 3):

32 $$\begin{aligned} \varOmega _k \cap L_k^- = \varOmega _k = \lbrace {x \in \mathbb {E} : {\Vert x - x_k \Vert }_{G_k} \le R_k} \rbrace , \qquad k \ge 0. \end{aligned}$$

Observe that, in this example, $\varGamma _k \equiv \sum _{i=0}^{k-1} a_i {\Vert {g_i} \Vert }_* = 0$ for all $k \ge 1$, so there is no preliminary semicertificate and the sliding gap is undefined. However, we can still ensure the convergence to zero of a certain meaningful measure of optimality, namely, the *average radius* of the localizers $\varOmega _k$:

33 $$\begin{aligned} {{\,\mathrm{avrad}\,}}\varOmega _k :=({{\,\mathrm{vol}\,}}\varOmega _k)^{1/n}, \qquad k \ge 0. \end{aligned}$$

Indeed, let us define the following functions for any real $c, p > 0$:

34 $$\begin{aligned} q_c(\gamma ) :=1 + \frac{c \gamma ^2}{2 (1 + \gamma )}, \qquad \zeta _{p, c}(\gamma ) :=\frac{[q_c(\gamma )]^p}{1 + \gamma }, \qquad \gamma > 0. \end{aligned}$$

According to Lemma 4, for any $k \ge 0$, we have

35 $$\begin{aligned} R_k^2 = [q_{1/2}(\gamma )]^k R^2. \end{aligned}$$

At the same time, in view of (18) and (26), $\det G_k = \prod _{i=0}^{k-1} (1 + b_i \Vert {g_i}\Vert _{G_i}^2) = (1 + \gamma )^k$ for all $k \ge 0$. Combining this with (32)–(34), we obtain, for any $k \ge 0$, that

36 $$\begin{aligned} {{\,\mathrm{avrad}\,}}\varOmega _k = \frac{R_k}{(\det G_k)^{1 / (2 n)}} = \frac{[q_{1/2}(\gamma )]^{k/2} R}{(1 + \gamma )^{k / (2 n)}} = [\zeta _{n, 1/2}(\gamma )]^{k / (2 n)} R. \end{aligned}$$

Let us now choose $\gamma$ which minimizes ${{\,\mathrm{avrad}\,}}\varOmega _k$. For such computations, the following auxiliary result is useful (see Sect. A for the proof).

#### Lemma 6

For any $c \ge 1/2$ and any $p \ge 2$, the function $\zeta _{p, c}$, defined in (34), attains its minimum at a unique point

37 $$\begin{aligned} \gamma _c(p) :=\frac{2}{\sqrt{c^2 p^2 - (2 c - 1)} + c p - 1} \in \left[ {\frac{1}{c p}}{\frac{2}{cp}}\right] \end{aligned}$$

with the corresponding value $\zeta _{p, c}\bigl ( \gamma _c(p) \bigr ) \le e^{-1 / (2 c p)}$.

Applying Lemma 6 to 36, we see that the optimal value of $\gamma$ is

38 $$\begin{aligned} \gamma :=\gamma _{1/2}(n) = \frac{2}{n/2 + n/2 - 1} = \frac{2}{n-1}, \end{aligned}$$

for which $\zeta _{n, 1/2}(\gamma ) \le e^{-1/n}$. With this choice of $\gamma$, we obtain, for all $k \ge 0$, that

39 $$\begin{aligned} {{\,\mathrm{avrad}\,}}\varOmega _k \le e^{-k / (2 n^2)} R. \end{aligned}$$

One can check that Algorithm 1 with parameters (26), (31) and (38) is, in fact, the standard Ellipsoid Method (see Remark 6).

### Ellipsoid method with preliminary semicertificate

As we have seen, we cannot measure the convergence rate of the standard Ellipsoid Method using the sliding gap because there is no preliminary semicertificate in this method. Let us present a modification of the standard Ellipsoid Method which does not have this drawback but still enjoys the same convergence rate as the original method (up to some absolute constants).

For this, let us choose the coefficients in the following way:

40 $$\begin{aligned} \alpha _k :=0, \qquad \theta :=\sqrt{2} - 1 \ (\approx 0.41), \qquad \gamma > 0. \end{aligned}$$

Then, in view of Lemma 4, for all $k \ge 0$, we have

41 $$\begin{aligned} R_k^2 = [q_1(\gamma )]^k R^2, \end{aligned}$$

Also, by Lemma 5, $\varGamma _k \ge \frac{1}{2} \theta R \sqrt{\gamma n [ (1 + \gamma )^{k/n} - 1 ]}$ for all $k \ge 1$. Thus, for each $k \ge 1$, we obtain the following estimate for the sliding gap (see (25)):

42 $$\begin{aligned} \varDelta _k \le \frac{[q_1(\gamma )]^k R}{ \theta \sqrt{\gamma n [ (1 + \gamma )^{k/n} - 1 ]}} = \frac{1}{\theta \kappa _k(\gamma , n)} [\zeta _{2 n, 1}(\gamma )]^{k/(2 n)} R, \end{aligned}$$

where $\kappa _k(\gamma , n) :=\sqrt{\gamma n ( 1 - \frac{1}{(1 + \gamma )^{k/n}})}$ and $\zeta _{2 n, 1}(\gamma )$ is defined in (34).

Note that the main factor in estimate (42) is $[\zeta _{2 n, 1}(\gamma )]^{k / (2 n)}$. Let us choose $\gamma$ by minimizing this expression. Applying Lemma 6, we obtain

43 $$\begin{aligned} \gamma :=\gamma _1(2 n) \in \left[ {\frac{1}{2 n}}{\frac{1}{n}}\right] . \end{aligned}$$

#### Theorem 1

In Algorithm 1 with parameters (26), (40), (43), for all $k \ge 1$,

$$\begin{aligned} \varDelta _k \le 6 e^{-k / (8 n^2)} R. \end{aligned}$$

#### Proof

Suppose $k \ge n^2$. According to Lemma 6, we have $\zeta _{2 n, 1}(\gamma ) \le e^{-1 / (4 n)}$. Hence, by (42), $\varDelta _k \le \frac{1}{\theta \kappa _k(\gamma , n)} e^{-k / (8 n^2)} R$. It remains to estimate from below $\theta \kappa _k(\gamma , n)$.

Since $k \ge n^2$, we have $(1 + \gamma )^{k/n} \ge (1 + \gamma )^n \ge 1 + \gamma n$. Hence, $\kappa _k(\gamma , n) \ge \frac{\gamma n}{\sqrt{1 + \gamma n}}$. Note that the function $\tau \mapsto \frac{\tau }{\sqrt{1 + \tau }}$ is increasing on $\mathbb {R}_+$. Therefore, using (43), we obtain $\kappa _k(\gamma , n) \ge \frac{1 / 2}{\sqrt{1 + 1 / 2}} = \frac{1}{\sqrt{6}}$. Thus, $\theta \kappa _k(\gamma , n) \ge \frac{\sqrt{2} - 1}{\sqrt{6}} \ge \frac{1}{6}$ for our choice of $\theta$.

Now suppose $k \le n^2$. Then, $6 e^{-k / (8 n^2)} \ge 6 e^{-1/8} \ge 5$. Therefore, it suffices to prove that $\varDelta _k \le 5 R$ or, in view of (24), that $\langle {g_i, x_i - x} \rangle \le 5 R {\Vert {g_i} \Vert }_*$, where $x \in \varOmega _k \cap L_k^-$ and $0 \le i \le k-1$ are arbitrary. Note that $\langle {g_i, x_i - x} \rangle \le {\Vert g_i \Vert }_{G_i}^* {\Vert x_i - x \Vert }_{G_i} \le {\Vert {g_i} \Vert }_* {\Vert x_i - x \Vert }_{G_i}$ since $G_i \succeq B$ (see (18)). Hence, it remains to prove that ${\Vert x_i - x \Vert }_{G_i} \le 5 R$.

Recall from (18) and (19) that $G_i \preceq G_k$ and $R_i \le R_k$. Therefore,

$$\begin{aligned} \begin{aligned} {\Vert x_i - x \Vert }_{G_i}&\le {\Vert x_i - x^* \Vert }_{G_i} + {\Vert x^* - x \Vert }_{G_i} \le {\Vert x_i - x^* \Vert }_{G_i} + {\Vert x^* - x \Vert }_{G_k} \\&\le {\Vert x_i - x^* \Vert }_{G_i} + {\Vert x_k - x^* \Vert }_{G_k} + {\Vert x_k - x \Vert }_{G_k} \le R_i + 2 R_k \le 3 R_k, \end{aligned} \end{aligned}$$

where the penultimate inequality follows from Lemma 2 and 3. According to (41), $R_k = [q_1(\gamma )]^{k/2} R \le [q_1(\gamma )]^{n^2/2} R$ (recall that $q_1(\gamma ) \ge 1$). Thus, it remains to show that $3 [q_1(\gamma )]^{n^2/2} \le 5$. But this is immediate. Indeed, by (34) and (43), we have $[q_1(\gamma )]^{n^2/2} \le e^{n^2 \gamma ^2 / (4 (1 + \gamma ))} \le e^{1/4}$, so $3 [q_1(\gamma )]^{n^2/2} \le 3 e^{1/4} \le 5$. $\square$

### Subgradient ellipsoid method

The previous algorithm still shares the drawback of the original Ellipsoid Method, namely, it does not work when $n \rightarrow \infty$. To eliminate this drawback, let us choose $\alpha _k$ similarly to how this is done in the Subgradient Method.

Consider the following choice of parameters:

44 $$\begin{aligned} \alpha _i :=\beta _i \sqrt{\frac{\theta }{\theta + 1}}, \qquad \theta :=\root 3 \of {2} - 1 \ (\approx 0.26), \qquad \gamma :=\gamma _1(2 n) \in \left[ {\frac{1}{2 n}}{\frac{1}{n}}\right] , \end{aligned}$$

where $\beta _i > 0$ are certain coefficients (to be specified later) and $\gamma _1(2 n)$ is defined in (37).

#### Theorem 2

In Algorithm 1 with parameters (26) and (44), where $\beta _0 \ge 1$, we have, for all $k \ge 1$,

45 $$\begin{aligned} \varDelta _k \le {\left\{ \begin{array}{ll} \frac{2}{\sum _{i=0}^{k-1} \beta _i} ( 1 + \sum _{i=0}^{k-1} \beta _i^2 ) R, &{} \text {if}\, k \le n^2, \\ 6 e^{-k / (8 n^2)} ( 1 + \sum _{i=0}^{k-1} \beta _i^2 ) R, &{} \text {if}\, k \ge n^2. \end{array}\right. } \end{aligned}$$

#### Proof

Applying Lemma 4 with $\tau :=\theta$ and using (44), we obtain

46 $$\begin{aligned} R_k^2 \le [q_1(\gamma )]^k C_k R^2, \qquad C_k = 1 + \sum _{i=0}^{k-1} \beta _i^2. \end{aligned}$$

At the same time, by Lemma 5, we have

47 $$\begin{aligned} \varGamma _k \ge R \Bigl ( \sqrt{\frac{\theta }{\theta + 1}} \sum _{i=0}^{k-1} \beta _i + \tfrac{1}{2} \theta \sqrt{\gamma n [(1 + \gamma )^{k/n} - 1]} \, \Bigr ). \end{aligned}$$

Note that $\frac{1}{2} \theta \sqrt{\gamma n} \le \frac{1}{2} \theta \le \sqrt{\theta / (\theta + 1)}$ by (44). Since $\beta _0 \ge 1$, we thus obtain

48 $$\begin{aligned} \begin{aligned} \varGamma _k&\ge \tfrac{1}{2} R \theta \sqrt{\gamma n} \Bigl ( 1 + \sqrt{(1 + \gamma )^{k/n} - 1} \, \Bigr ) \ge \tfrac{1}{2} R \theta \sqrt{\gamma n} (1 + \gamma )^{k / (2 n)} \\&\ge \tfrac{1}{2 \sqrt{2}} R \theta (1 + \gamma )^{k / (2 n)} \ge \tfrac{1}{12} R (1 + \gamma )^{k / (2 n)}, \end{aligned} \end{aligned}$$

where the last two inequalities follow from (44). Therefore, by (25), (46) and (48),

$$\begin{aligned} \varDelta _k \le \frac{R_k^2}{2 \varGamma _k} \le 6 \frac{[q_1(\gamma )]^k}{(1 + \gamma )^{k/(2n)}} C_k R = 6 [\zeta _{2 n, 1}(\gamma )]^{k/(2n)} C_k R, \end{aligned}$$

where $\zeta _{2n, 1}(\gamma )$ is defined in (34). Observe that, for our choice of $\gamma$, by Lemma 6, we have $\zeta _{2 n, 1}(\gamma ) \le e^{-1 / (4 n)}$. This proves the second estimate^3^ in (45).

On the other hand, dropping the second term in (47), we can write

49 $$\begin{aligned} \varGamma _k \ge R \sqrt{\frac{\theta }{\theta + 1}} \sum _{i=0}^{k-1} \beta _i. \end{aligned}$$

Suppose $k \le n^2$. Then, from (34) and (44), it follows that

$$\begin{aligned}{}[q_1(\gamma )]^k \le [q_1(\gamma )]^{n^2} \le e^{\gamma ^2 n^2 / (2 (1 + \gamma ))} \le \sqrt{e}. \end{aligned}$$

Hence, by (46), $R_k \le \sqrt{e} C_k R^2$. Combining this with (25) and (49), we obtain

$$\begin{aligned} \varDelta _k \le \frac{1}{2} \sqrt{\frac{e (\theta + 1)}{\theta }} \frac{1}{\sum _{i=0}^{k-1} \beta _i} C_k R. \end{aligned}$$

By numerical evaluation, one can verify that, for our choice of $\theta$, we have $\frac{1}{2} \sqrt{\frac{e (\theta + 1)}{\theta }} \le 2$. This proves the first estimate in (45). $\square$

Exactly as in the Subgradient Method, we can use the following two strategies for choosing the coefficients $\beta _i$:

1. We fix in advance the number of iterations $k \ge 1$ of the method and use constant coefficients $\beta _i :=\frac{1}{\sqrt{k}}$, $0 \le i \le k-1$. In this case,
  50 $$\begin{aligned} \varDelta _k \le {\left\{ \begin{array}{ll} 4 R / \sqrt{k}, &{} \text {if}\, k \le n^2, \\ 12 R e^{-k / (8 n^2)}, &{} \text {if}\, k \ge n^2. \end{array}\right. } \end{aligned}$$
2. We use time-varying coefficients $\beta _i :=\frac{1}{\sqrt{i + 1}}$, $i \ge 0$. In this case,
  $$\begin{aligned} \varDelta _k \le {\left\{ \begin{array}{ll} 2 (\ln k + 2) R / \sqrt{k}, &{} \text {if}\, k \le n^2, \\ 6 (\ln k + 2) R e^{-k / (8 n^2)}, &{} \text {if}\, k \ge n^2. \end{array}\right. } \end{aligned}$$

Let us discuss convergence rate estimate (50). Up to absolute constants, this estimate is exactly the same as in the Subgradient Method when $k \le n^2$ and as in the Ellipsoid Method when $k \ge n^2$. In particular, when $n \rightarrow \infty$, we recover the convergence rate of the Subgradient Method.

To provide a better interpretation of the obtained results, let us compare the convergence rates of the Subgradient and Ellipsoid methods:

$$\begin{aligned} \begin{aligned} \text {Subgradient Method:}&\qquad 1 / \sqrt{k} \\ \text {Ellipsoid Method:}&\qquad e^{-k / (2 n^2)}. \end{aligned} \end{aligned}$$

To compare these rates, let us look at their squared ratio:

$$\begin{aligned} \rho _k :=\Bigl ( \frac{1/\sqrt{k}}{e^{-k/(2 n^2)}} \Bigr )^2 = \frac{e^{k / n^2}}{k}. \end{aligned}$$

Let us find out for which values of *k* the rate of the Subgradient Method is better than that of the Ellipsoid Method and vice versa. We assume that $n \ge 2$.

Note that the function $\tau \mapsto e^{\tau } / \tau$ is strictly decreasing on $\left( {0, 1} \right]$ and strictly increasing on $\left[ {1, +\infty } \right)$ (indeed, its derivative equals $e^\tau (\tau - 1) / \tau ^2$). Hence, $\rho _k$ is strictly decreasing in *k* for $1 \le k \le n^2$ and strictly increasing in *k* for $k \ge n^2$. Since $n \ge 2$, we have $\rho _2 = e^{2 / n^2} / 2 \le e^{1/2} / 2 \le 1$. At the same time, $\rho _k \rightarrow +\infty$ when $k \rightarrow \infty$. Therefore, there exists a unique integer $K_0 \ge 2$ such that $\rho _k \le 1$ for all $k \le K_0$ and $\rho _k \ge 1$ for all $k \ge K_0$.

Let us estimate $K_0$. Clearly, for any $n^2 \le k \le n^2 \ln (2 n)$, we have

$$\begin{aligned} \rho _k \le \frac{e^{n^2 \ln (2 n) / n^2}}{n^2 \ln (2 n)} = \frac{2}{n \ln (2 n)} \le 1, \end{aligned}$$

while, for any $k \ge 3 n^2 \ln (2 n)$, we have

$$\begin{aligned} \rho _k \ge \frac{e^{3 n^2 \ln (2 n)} / n^2}{3 n^2 \ln (2 n)} = \frac{(2 n)^3}{3 n^2 \ln (2 n)} = \frac{8 n}{3 \ln (2 n)} \ge 1. \end{aligned}$$

Hence,

$$\begin{aligned} n^2 \ln (2 n) \le K_0 \le 3 n^2 \ln (2 n). \end{aligned}$$

Thus, up to an absolute constant, $n^2 \ln (2 n)$ is the switching moment, starting from which the rate of the Ellipsoid Method becomes better than that of the Subgradient Method.

Returning to our obtained estimate (50), we see that, ignoring absolute constants and ignoring the “small” region of the values of *k* between $n^2$ and $n^2 \ln n$, our convergence rate is basically the best of the corresponding convergence rates of the Subgradient and Ellipsoid methods.

## Constructing accuracy semicertificate

Let us show how to convert a preliminary accuracy semicertificate, produced by Algorithm 1, into a semicertificate whose gap on the initial solid is upper bounded by the sliding gap. The key ingredient here is the following auxiliary algorithm which was first proposed in [16] for building accuracy certificates in the standard Ellipsoid Method.

### Augmentation algorithm

Let $k \ge 0$ be an integer and let $Q_0, \dots , Q_k$ be solids in $\mathbb {E}$ such that

51 $$\begin{aligned} \hat{Q}_i :=\lbrace {x \in Q_i : \langle {g_i, x - x_i} \rangle \le 0} \rbrace \subseteq Q_{i+1}, \qquad 0 \le i \le k-1, \end{aligned}$$

where $x_i \in \mathbb {E}$, $g_i \in \mathbb {E}^*$. Further, suppose that, for any $s \in \mathbb {E}^*$ and any $0 \le i \le k-1$, we can compute a *dual multiplier* $\mu \ge 0$ such that

52 $$\begin{aligned} \max _{x \in \hat{Q}_i} \langle {s, x} \rangle = \max _{x \in Q_i} [ \langle {s, x} \rangle + \mu \langle {g_i, x_i - x} \rangle ] \end{aligned}$$

(provided that certain regularity conditions hold). Let us abbreviate any solution $\mu$ of this problem by $\mu (s, Q_i, x_i, g_i)$.

Consider now the following routine. 
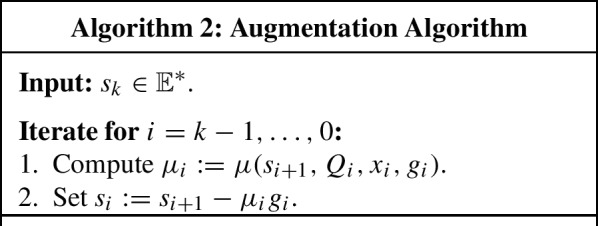


#### Lemma 7

Let $\mu _0, \dots , \mu _{k-1} \ge 0$ be generated by Algorithm 2. Then,

$$\begin{aligned} \max _{x \in Q_0} \Bigl [ \langle {s_k, x} \rangle + \sum _{i=0}^{k-1} \mu _i \langle {g_i, x_i - x} \rangle \Bigr ] \le \max _{x \in Q_k} \langle {s_k, x} \rangle . \end{aligned}$$

#### Proof

Indeed, at every iteration $i = k-1, \dots , 0$, we have

$$\begin{aligned} \begin{aligned} \max _{x \in Q_{i+1}} \langle {s_{i+1}, x} \rangle&\ge \max _{x \in \hat{Q}_i} \langle {s_{i+1}, x} \rangle = \max _{x \in Q_i} [ \langle {s_{i+1}, x} \rangle + \mu _i \langle {g_i, x_i - x} \rangle ] \\&= \max _{x \in Q_i} \langle {s_i, x} \rangle + \mu _i \langle {g_i, x_i} \rangle . \end{aligned} \end{aligned}$$

Summing up these inequalities for $i = 0, \dots , k-1$, we obtain

$$\begin{aligned} \max _{x \in Q_k} \langle {s_k, x} \rangle \ge \max _{x \in Q_0} \langle {s_0, x} \rangle + \sum _{i=0}^{k-1} \mu _i \langle {g_i, x_i} \rangle = \max _{x \in Q_0} \Bigl [ \langle {s_k, x} \rangle + \sum _{i=0}^{k-1} \langle {g_i, x_i - x} \rangle \Bigr ], \end{aligned}$$

where the identity follows from the fact that $s_0 = s_k - \sum _{i=0}^{k-1} \mu _i g_i$. $\square$

### Methods with preliminary certificate

Let us apply the Augmentation Algorithm for building an accuracy semicertificate for Algorithm 1. We only consider those instances for which $\varGamma _k :=\sum _{i=0}^{k-1} a_i {\Vert {g_i} \Vert }_* > 0$ so that the sliding gap $\varDelta _k$ is well-defined:

$$\begin{aligned} \begin{aligned} \varDelta _k :=\max _{x \in \varOmega _k} \frac{1}{\varGamma _k} [ -\ell _k(x) ]&= \max _{x \in \varOmega _k \cap L_k^-} \frac{1}{\varGamma _k} [ -\ell _k(x) ] \\&= \max _{x \in \varOmega _k \cap L_k^-} \frac{1}{\varGamma _k} \sum _{i=0}^{k-1} a_i \langle {g_i, x_i - x} \rangle . \end{aligned} \end{aligned}$$

Recall that the vector $a :=(a_0, \dots , a_{k-1})$ is called a preliminary semicertificate.

For technical reasons, it will be convenient to add the following termination criterion into Algorithm 1:

53 $$\begin{aligned} \text { Terminate Algorithm} \, {1} \text { at Step}~2~\text {if}~U_k \le \delta {\Vert {g_k} \Vert }_*, \end{aligned}$$

where $\delta > 0$ is a fixed constant. Depending on whether this termination criterion has been satisfied at iteration *k*, we call it a *terminal* or *nonterminal* iteration, respectively.

#### Remark 5

In practice, one can set $\delta$ to an arbitrarily small value (within machine precision) if the desired target accuracy is unknown. As can be seen from the subsequent discussion, the main purpose of the termination criterion (53) is to ensure that $U_k$ never becomes equal to zero during the iterations of Algorithm 1. This guarantees the existence of dual multiplier in (52) for any $s \in \mathbb {E}^*$ at every nonterminal iteration. The case $U_k = 0$ corresponds to the degenerate situation when Algorithm 1 has “accidentally” found an exact solution.

Let $k \ge 1$ be an iteration of Algorithm 1. According to Lemma 2, the sets $Q_i :=\varOmega _i \cap L_i^-$ satisfy (51). Since the method has not been terminated during the course of the previous iterations, we have^4^$U_i > 0$ for all $0 \le i \le k-1$. Therefore, for any $0 \le i \le k-1$, there exists $x \in Q_i$ such that $\langle {g_i, x - x_i} \rangle < 0$. This guarantees the existence of dual multiplier in (52).

Let us apply Algorithm 2 to $s_k :=-\sum _{i=0}^{k-1} a_i g_i$ in order to obtain dual multipliers $\mu :=(\mu _0, \dots , \mu _{k-1})$. From Lemma 7, it follows that

$$\begin{aligned} \max _{x \in {B}\left( {x_0, R} \right) } \sum _{i=0}^{k-1} (a_i + \mu _i) \langle {g_i, x_i - x} \rangle \le \max _{x \in Q_k} \sum _{i=0}^{k-1} a_i \langle {g_i, x_i - x} \rangle = \varGamma _k \varDelta _k, \end{aligned}$$

(note that $Q_0 = \varOmega _0 \cap L_0^- = {B}\left( {x_0, R} \right)$). Thus, defining $\lambda :=a + \mu$, we obtain $\varGamma _k(\lambda ) \equiv \sum _{i=0}^{k-1} \lambda _i {\Vert {g_i} \Vert }_* \ge \sum _{i=0}^{k-1} a_i {\Vert {g_i} \Vert }_* \equiv \varGamma _k > 0$ and

$$\begin{aligned} \delta _k(\lambda ) \equiv \max _{x \in {B}\left( {x_0, R} \right) } \frac{1}{\varGamma _k(\lambda )} \sum _{i=0}^{k-1} \lambda _i \langle {g_i, x_i - x} \rangle \le \frac{\varGamma _k}{\varGamma _k(\lambda )} \varDelta _k \le \varDelta _k, \end{aligned}$$

Thus, $\lambda$ is a semicertificate whose gap on ${B}\left( {x_0, R} \right)$ is bounded by the sliding gap $\varDelta _k$.

If $k \ge 0$ is a terminal iteration, then, by the termination criterion and the definition of $U_k$ (see Algorithm 1), we have $\max _{x \in \varOmega _k \cap L_k^-} \frac{1}{{\Vert {g_k} \Vert }_*} \langle {g_k, x_k - x} \rangle \le \delta$. In this case, we apply Algorithm 2 to $s_k :=-g_k$ to obtain dual multipliers $\mu _0, \dots , \mu _{k-1}$. By the same reasoning as above but with the vector $(0, \dots , 0, 1)$ instead of $(a_0, \dots , a_{k-1})$, we can obtain that $\delta _{k+1}(\lambda ) \le \delta$, where $\lambda :=(\mu _0, \dots , \mu _{k-1}, 1)$.

### Standard ellipsoid method

In the standard Ellipsoid Method, there is no preliminary semicertificate. Therefore, we cannot apply the above procedure. However, in this method, it is still possible to generate an accuracy semicertificate, although the corresponding procedure is slightly more involved. Let us now briefly describe this procedure and discuss how it differs from the previous approach. For details, we refer the reader to [16].

Let $k \ge 1$ be an iteration of the method. There are two main steps. The first step is to find a direction $s_k$, in which the “width” of the ellipsoid $\varOmega _k$ (see (32)) is minimal:

$$\begin{aligned} s_k :=\mathop {\mathrm{argmin}}\limits _{{\Vert {s} \Vert }_* = 1} \max _{x, y \in \varOmega _k} \langle {s, x - y} \rangle = \mathop {\mathrm{argmin}}\limits _{{\Vert {s} \Vert }_* = 1} \bigl [ \, \max _{x \in \varOmega _k} \langle {s, x} \rangle - \min _{x \in \varOmega _k} \langle {s, x} \rangle \bigr ]. \end{aligned}$$

It is not difficult to see that $s_k$ is given by the unit eigenvector^5^ of the operator $G_k$, corresponding to the largest eigenvalue. For the corresponding minimal “width” of the ellipsoid, we have the following bound via the average radius:

54 $$\begin{aligned} \max _{x, y \in \varOmega _k} \langle {s_k, x - y} \rangle \le \rho _k, \end{aligned}$$

where $\rho _k :=2 {{\,\mathrm{avrad}\,}}\varOmega _k$. Recall that ${{\,\mathrm{avrad}\,}}\varOmega _k \le e^{-k / (2 n^2)} R$ in view of (39).

At the second step, we apply Algorithm 2 two times with the sets $Q_i :=\varOmega _i$: first, to the vector $s_k$ to obtain dual multipliers $\mu :=(\mu _0, \dots , \mu _{k-1})$ and then to the vector $-s_k$ to obtain dual multipliers $\mu ' :=(\mu _0', \dots , \mu _{k-1}')$. By Lemma 7 and (54), we have

$$\begin{aligned} \begin{aligned} \max _{x \in {B}\left( {x_0, R} \right) } \Bigl [ \langle {s_k, x - x_k} \rangle + \sum _{i=0}^{k-1} \mu _i \langle {g_i, x_i - x} \rangle \Bigr ]&\le \max _{x \in \varOmega _k} \langle {s_k, x - x_k} \rangle \le \rho _k, \\ \max _{x \in {B}\left( {x_0, R} \right) } \Bigl [ \langle {s_k, x_k - x} \rangle + \sum _{i=0}^{k-1} \mu _i' \langle {g_i, x_i - x} \rangle \Bigr ]&\le \max _{x \in \varOmega _k} \langle {s_k, x_k - x} \rangle \le \rho _k \end{aligned} \end{aligned}$$

(note that $Q_0 = \varOmega _0 = {B}\left( {x_0, R} \right)$). Consequently, for $\lambda :=\mu + \mu '$, we obtain

$$\begin{aligned} \max _{x \in {B}\left( {x_0, R} \right) } \sum _{i=0}^{k-1} \lambda _i \langle {g_i, x_i - x} \rangle \le 2 \rho _k. \end{aligned}$$

Finally, one can show that

$$\begin{aligned} \varGamma _k(\lambda ) \equiv \sum _{i=0}^{k-1} \lambda _i {\Vert {g_i} \Vert }_* \ge \frac{r - \rho _k}{D}, \end{aligned}$$

where *D* is the diameter of *Q* and *r* is the maximal of the radii of Euclidean balls contained in *Q*. Thus, whenever $\rho _k < r$, $\lambda$ is a semicertificate with the following gap on ${B}\left( {x_0, R} \right)$:

$$\begin{aligned} \delta _k(\lambda ) \equiv \max _{x \in {B}\left( {x_0, R} \right) } \frac{1}{\varGamma _k(\lambda )} \sum _{i=0}^{k-1} \lambda _i \langle {g_i, x_i - x} \rangle \le \frac{2 \rho _k D}{r - \rho _k}. \end{aligned}$$

Compared to the standard Ellipsoid Method, we see that, in the Subgradient Ellipsoid methods, the presence of the preliminary semicertificate removes the necessity in finding the minimal-“width” direction and requires only one run of the Augmentation Algorithm.

## Implementation details

### Explicit representations

In the implementation of Algorithm 1, instead of the operators $G_k$, it is better to work with their inverses $H_k :=G_k^{-1}$. Applying the Sherman-Morrison formula to (18), we obtain the following update rule for $H_k$:

55 $$\begin{aligned} H_{k+1} = H_k - \frac{b_k H_k g_k g_k^*H_k}{ 1 + b_k \langle {g_k, H_k g_k} \rangle }, \quad k \ge 0. \end{aligned}$$

Let us now obtain an explicit formula for the next test point $x_{k+1}$. This has already been partly done in the proof of Lemma 3. Indeed, recall that $x_{k+1}$ is the minimizer of the function $\psi _{k+1}(x)$. From (21), we see that $x_{k+1} = x_k - (a_k + \frac{1}{2} b_k U_k) H_{k+1} g_k$. Combining it with (55), we obtain

56 $$\begin{aligned} x_{k+1} = x_k - \frac{a_k + \frac{1}{2} b_k U_k}{ 1 + b_k \langle {g_k, H_k g_k} \rangle } H_k g_k, \quad k \ge 0. \end{aligned}$$

Finally, one can obtain the following explicit representations for $L_k^-$ and $\varOmega _k$:

57 $$\begin{aligned} L_k^- = \lbrace {x \in \mathbb {E} : \langle {c_k, x} \rangle \le \sigma _k} \rbrace , \qquad \varOmega _k = \lbrace {x \in \mathbb {E} : {\Vert x - z_k \Vert }_{H_k^{-1}}^2 \le D_k} \rbrace , \end{aligned}$$

where, for any $k \ge 0$,

58 $$\begin{aligned} \begin{aligned} c_0 :=0, \quad \sigma _0 :=0, \qquad c_{k+1} :=c_k + a_k g_k, \quad \sigma _{k+1} :=\sigma _k + a_k \langle {g_k, x_k} \rangle , \\ z_k :=x_k - H_k c_k, \quad D_k :=R_k^2 + 2 (\sigma _k - \langle {c_k, x_k} \rangle ) + \langle {c_k, H_k c_k} \rangle . \end{aligned} \end{aligned}$$

Indeed, recalling the definition of functions $\ell _k$, we see that $\ell _k(x) = \langle {c_k, x} \rangle - \sigma _k$ for all $x \in \mathbb {E}$. Therefore, $L_k^- \equiv \lbrace {x : \ell _k(x) \le 0} \rbrace = \lbrace {x : \langle {c_k, x} \rangle \le \sigma _k} \rbrace$. Further, by Lemma 3, $\varOmega _k = \lbrace {x : \langle {c_k, x} \rangle + \frac{1}{2} {\Vert x - x_k \Vert }_{G_k}^2 \le \frac{1}{2} R_k^2 + \sigma _k} \rbrace$. Note that $\langle {c_k, x} \rangle + \frac{1}{2} {\Vert x - x_k \Vert }_{G_k}^2 = \frac{1}{2} {\Vert x - z_k \Vert }_{G_k}^2 + \langle {c_k, x_k} \rangle - \frac{1}{2} \Vert {c_k}\Vert _{G_k}^2$ for any $x \in \mathbb {E}$. Hence, $\varOmega _k = \lbrace {x : \frac{1}{2} {\Vert x - z_k \Vert }_{G_k}^2 \le \frac{1}{2} D_k} \rbrace$.

#### Remark 6

Now we can justify the claim made in Sect. 4.2 that Algorithm 1 with parameters (26), (31) and (38) is the standard Ellipsoid Method. Indeed, from (26) and (32), we see that $b_k = \frac{\gamma }{\langle {g_k, H_k g_k} \rangle }$ and $U_k = R_k \langle {g_k, H_k g_k} \rangle ^{1/2}$. Also, in view of (38), $\frac{\gamma }{1 + \gamma } = \frac{2}{n + 1}$. Hence, by (56) and (55),

59 $$\begin{aligned} \begin{aligned} x_{k+1}&= x_k - \frac{R_k}{n + 1} \frac{H_k g_k}{\langle {g_k, H_k g_k} \rangle ^{1/2}}, \\ H_{k+1}&= H_k - \frac{2}{n + 1} \frac{H_k g_k g_k^*H_k}{\langle {g_k, H_k g_k} \rangle }, \qquad k \ge 0. \end{aligned} \end{aligned}$$

Further, according to (35) and (38), for any $k \ge 0$, we have $R_k^2 = q^k R^2$, where $q = 1 + \frac{1}{(n - 1) (n + 1)} = \frac{n^2}{n^2 - 1}$. Thus, method (59) indeed coincides^6^ with the standard Ellipsoid Method (2) under the change of variables $W_k :=R_k^2 H_k$.

### Computing support function

To calculate $U_k$ in Algorithm 1, we need to compute the following quantity (see (57)):

$$\begin{aligned} U_k = \max _x \lbrace {\langle {g_k, x_k - x} \rangle : {\Vert x - z_k \Vert }_{H_k^{-1}}^2 \le D_k, \langle {c_k, x} \rangle \le \sigma _k} \rbrace . \end{aligned}$$

Let us discuss how to do this.

First, let us introduce the following support function to simplify our notation:

$$\begin{aligned} \xi (H, s, a, \beta ) :=\max _x \lbrace {\langle {s, x} \rangle : {\Vert x \Vert }_{H^{-1}}^2 \le 1, \langle {a, x} \rangle \le \beta } \rbrace , \end{aligned}$$

where $H :\mathbb {E}^* \rightarrow \mathbb {E}$ is a self-adjoint positive definite linear operator, $s, a \in \mathbb {E}^*$ and $\beta \in \mathbb {R}$. In this notation, assuming that $D_k > 0$, we have

$$\begin{aligned} U_k = \langle {g_k, x_k - z_k} \rangle + \xi (D_k H_k, -g_k, c_k, \sigma _k - \langle {c_k, z_k} \rangle ). \end{aligned}$$

Let us show how to compute $\xi (H, s, a, \beta )$. Dualizing the linear constraint, we obtain

60 $$\begin{aligned} \xi (H, s, a, \beta ) = \min _{\tau \ge 0} \bigl [ {\Vert s - \tau a \Vert }_{H^{-1}}^* + \tau \beta \bigr ], \end{aligned}$$

provided that there exists some $x \in \mathbb {E}$ such that ${\Vert x \Vert }_{H^{-1}} < 1$, $\langle {a, x} \rangle \le \beta$ (Slater condition). One can show that (60) has the following solution (see Lemma 10):

61 $$\begin{aligned} \tau (H, s, a, \beta ) :={\left\{ \begin{array}{ll} 0, &{} \text {if}\, \langle {a, H s} \rangle \le \beta {\Vert s \Vert }_{H^{-1}}^*, \\ u(H, s, a, \beta ), &{} \text {otherwise}, \end{array}\right. } \end{aligned}$$

where $u(H, s, a, \beta )$ is the unconstrained minimizer of the objective function in (60).

Let us present an explicit formula for $u(H, s, a, \beta )$. For future use, it will be convenient to write down this formula in a slightly more general form for the following multidimensional^7^ variant of problem (60):

62 $$\begin{aligned} \min _{u \in \mathbb {R}^m} \bigl [ {\Vert s - A u \Vert }_{H^{-1}}^* + \langle {u, b} \rangle \bigr ], \end{aligned}$$

where $s \in \mathbb {E}^*$, $H :\mathbb {E}^* \rightarrow \mathbb {E}$ is a self-adjoint positive definite linear operator, $A :\mathbb {R}^m \rightarrow \mathbb {E}^*$ is a linear operator with trivial kernel and $b \in \mathbb {R}^m$, $\langle {b, (A^*H A)^{-1} b} \rangle < 1$. It is not difficult to show that problem (62) has the following unique solution (see Lemma 9):

63 $$\begin{aligned}&u(H, s, A, b) :=(A^*H A)^{-1} (A^*s - r b),\nonumber \\&r :=\sqrt{\frac{ \langle {s, H s} \rangle - \langle {s, A (A^*H A)^{-1} A^*s} \rangle }{1 - \langle {b, (A^*H A)^{-1} b} \rangle }}. \end{aligned}$$

Note that, in order for the above approach to work, we need to guarantee that the sets $\varOmega _k$ and $L_k^-$ satisfy a certain regularity condition, namely, ${{\,\mathrm{int}\,}}\varOmega _k \cap L_k^- \ne \emptyset$. This condition can be easily fulfilled by adding into Algorithm 1 the termination criterion (53).

#### Lemma 8

Consider Algorithm 1 with termination criterion (53). Then, at each iteration $k \ge 0$, at the beginning of Step , we have ${{\,\mathrm{int}\,}}\varOmega _k \cap L_k^- \ne \emptyset$. Moreover, if *k* is a nonterminal iteration, we also have $\langle {g_k, x - x_k} \rangle \le 0$ for some $x \in {{\,\mathrm{int}\,}}\varOmega _k \cap L_k^-$.

#### Proof

Note that ${{\,\mathrm{int}\,}}\varOmega _0 \cap L_0^- = {{\,\mathrm{int}\,}}{B}\left( {x_0, R} \right) \ne \emptyset$. Now suppose ${{\,\mathrm{int}\,}}\varOmega _k \cap L_k^- \ne \emptyset$ for some nonterminal iteration $k \ge 0$. Denote $P_k^- :=\lbrace {x \in \mathbb {E} : \langle {g_k, x - x_k} \rangle \le 0} \rbrace$. Since iteration *k* is nonterminal, $U_k > 0$ and hence $\varOmega _k \cap L_k^- \cap {{\,\mathrm{int}\,}}P_k^- \ne \emptyset$. Combining it with the fact that ${{\,\mathrm{int}\,}}\varOmega _k \cap L_k^- \ne \emptyset$, we obtain ${{\,\mathrm{int}\,}}\varOmega _k \cap L_k^- \cap {{\,\mathrm{int}\,}}P_k^- \ne \emptyset$ and, in particular, ${{\,\mathrm{int}\,}}\varOmega _k \cap L_k^- \cap P_k^- \ne \emptyset$. At the same time, slightly modifying the proof of Lemma 2 (using that ${{\,\mathrm{int}\,}}\varOmega _i = \lbrace {x \in \mathbb {E} : \omega _i(x) < \frac{1}{2} R^2} \rbrace$ for any $i \ge 0$ since $\omega _i$ is a strictly convex quadratic function), it is not difficult to show that ${{\,\mathrm{int}\,}}\varOmega _k \cap L_k^- \cap P_k^- \subseteq {{\,\mathrm{int}\,}}\varOmega _{k+1} \cap L_{k+1}^-$. Thus, ${{\,\mathrm{int}\,}}\varOmega _{k+1} \cap L_{k+1}^- \ne \emptyset$, and we can continue by induction. $\square$

### Computing dual multipliers

Recall from Sect. 5 that the procedure for generating an accuracy semicertificate for Algorithm 1 requires one to repeatedly carry out the following operation: given $s \in \mathbb {E}^*$ and some iteration number $i \ge 0$, compute a dual multiplier $\mu \ge 0$ such that

$$\begin{aligned} \max _{x \in \varOmega _i \cap L_i^-} \lbrace {\langle {s, x} \rangle : \langle {g_i, x - x_i} \rangle \le 0} \rbrace = \max _{x \in \varOmega _i \cap L_i^-} \bigl [ \langle {s, x} \rangle + \mu \langle {g_i, x_i - x} \rangle \bigr ]. \end{aligned}$$

This can be done as follows.

First, using (57), let us rewrite the above primal problem more explicitly:

$$\begin{aligned} \max _x \lbrace {\langle {s, x} \rangle : {\Vert x - z_i \Vert }_{H_i^{-1}}^2 \le D_i, \langle {c_i, x} \rangle \le \sigma _i, \langle {g_i, x - x_i} \rangle \le 0} \rbrace . \end{aligned}$$

Our goal is to dualize the second linear constraint and find the corresponding multiplier. However, for the sake of symmetry, it is better to dualize both linear constraints, find the corresponding multipliers and then keep only the second one.

Let us simplify our notation by introducing the following problem:

64 $$\begin{aligned} \max _x \lbrace {\langle {s, x} \rangle : {\Vert x \Vert }_{H^{-1}} \le 1, \langle {a_1, x} \rangle \le b_1, \langle {a_2, x} \rangle \le b_2} \rbrace , \end{aligned}$$

where $H :\mathbb {E}^* \rightarrow \mathbb {E}$ is a self-adjoint positive definite linear operator, $s, a_1, a_2 \in \mathbb {E}^*$ and $b_1, b_2 \in \mathbb {R}$. Clearly, our original problem can be transformed into this form by setting $H :=D_i H_i$, $a_1 :=c_i$, $a_2 :=g_i$, $b_1 :=\sigma _i - \langle {c_i, z_i} \rangle$, $b_2 :=\langle {g_i, x_i - z_i} \rangle$. Note that this transformation does not change the dual multipliers.

Dualizing the linear constraints in (64), we obtain the following dual problem:

65 $$\begin{aligned} \min _{\mu \in \mathbb {R}_+^2} \bigl [ {\Vert s - \mu _1 a_1 - \mu _2 a_2 \Vert }_{H^{-1}}^* + \mu _1 b_1 + \mu _2 b_2 \bigr ], \end{aligned}$$

which is solvable provided the following Slater condition holds:

66 $$\begin{aligned} \exists x \in \mathbb {E} :{\Vert x \Vert }_{H^{-1}} < 1, \langle {a_1, x} \rangle \le b_1, \langle {a_2, x} \rangle \le b_2. \end{aligned}$$

Note that (66) can be ensured by adding termination criterion (53) into Algorithm 1 (see Lemma 8).

A solution of (65) can be found using Algorithm 3. In this routine, $\tau (\cdot )$, $\xi (\cdot )$ and $u(\cdot )$ are the auxiliary operations, defined in Sect. 6.2, and $A :=(a_1, a_2)$ is the linear operator $A u :=u_1 a_1 + u_2 a_2$ acting from $\mathbb {R}^2$ to $\mathbb {E}^*$. The correctness of Algorithm 3 is proved in Theorem 3.
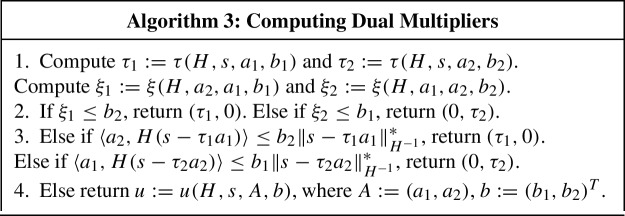


### Time and memory requirements

Let us discuss the time and memory requirements of Algorithm 1, taking into account the previously mentioned implementation details.

The main objects in Algorithm 1, which need to be stored and updated between iterations, are the test points $x_k$, matrices $H_k$, scalars $R_k$, vectors $c_k$ and scalars $\sigma _k$, see (19), (55), 56 and (58) for the corresponding updating formulas. To store all these objects, we need $O(n^2)$ memory.

Consider now what happens at each iteration *k*. First, we compute $U_k$. For this, we calculate $z_k$ and $D_k$ according to (58) and then perform the calculations described in Sect. 6.2. The most difficult operation there is computing the matrix-vector product, which takes $O(n^2)$ time. After that, we calculate the coefficients $a_k$ and $b_k$ according to (26), where $\alpha _k$, $\theta$ and $\gamma$ are certain scalars, easily computable for all main instances of Algorithm 1 (see Sects. 4.1–4.4). The most expensive step there is computing the norm ${\Vert g_k \Vert }_{G_k}^*$, which can be done in $O(n^2)$ operations by evaluating the product $H_k g_k$. Finally, we update our main objects, which takes $O(n^2)$ time.

Thus, each iteration of Algorithm 1 has $O(n^2)$ time and memory complexities, exactly as in the standard Ellipsoid Method.

Now let us analyze the complexity of the auxiliary procedure from Sect. 5 for converting a preliminary semicertificate into a semicertificate. The main operation in this procedure is running Algorithm 2, which iterates “backwards”, computing some dual multiplier $\mu _i$ at each iteration $i = k-1, \dots , 0$. Using the approach from Sect. 6.3, we can compute $\mu _i$ in $O(n^2)$ time, provided that the objects $x_i$, $g_i$, $H_i$, $z_i$, $D_i$, $c_i$, $\sigma _i$ are stored in memory. Note, however, that, in contrast to the “forward” pass, when iterating “backwards”, there is no way to efficiently recompute all these objects without storing in memory a certain “history” of the main process from iteration 0 up to *k*. The simplest choice is to keep in this “history” all the objects mentioned above, which requires $O(k n^2)$ memory. A slightly more efficient idea is to keep the matrix-vector products $H_i g_i$ instead of $H_i$ and then use (55) to recompute $H_i$ from $H_{i+1}$ in $O(n^2)$ operations. This allows us to reduce the size of the “history” down to $O(k n)$ while still keeping the $O(k n^2)$ total time complexity of the auxiliary procedure. Note that these estimates are exactly the same as those for the best currently known technique for generating accuracy certificates in the standard Ellipsoid Method [16]. In particular, if we generate a semicertificate only once at the very end, then the time complexity of our procedure is comparable to that of running the standard Ellipsoid Method without computing any certificates. Alternatively, as suggested in [16], one can generate semicertificates, say, every $2, 4, 8, 16, \dots$ iterations. Then, the total “overhead” of the auxiliary procedure for generating semicertificates will be comparable to the time complexity of the method itself.

## Conclusion

In this paper, we have addressed one of the issues of the standard Ellipsoid Method, namely, its poor convergence for problems of large dimension *n*. For this, we have proposed a new algorithm which can be seen as the combination of the Subgradient and Ellipsoid methods.

Our developments can be considered as a first step towards constructing universal methods for nonsmooth problems with convex structure. Such methods could significantly improve the practical efficiency of solving various applied problems.

Note that there are still some open questions. First, the convergence estimate of our method with time-varying coefficients contains an extra factor proportional to the logarithm of the iteration counter. We have seen that this logarithmic factor has its roots yet in the Subgradient Method. However, as discussed in Remark 4, for the Subgradient Method, this issue can be easily resolved by allowing projections onto the feasible set and working with “truncated” gaps. An even better alternative, which does not require any of this machinery, is to use Dual Averaging [18] instead of the Subgradient Method. It is an interesting question whether one can combine the Dual Averaging with the Ellipsoid Method similarly to how we have combined the Subgradient and Ellipsoid methods.

Second, the convergence rate estimate, which we have obtained for our method, is not continuous in the dimension *n*. Indeed, for small values of the iteration counter *k*, this estimate behaves as that of the Subgradient Method and then, at some moment (around $n^2$), it switches to the estimate of the Ellipsoid Method. As discussed at the end of Sect. 4.4, there exists some “small” gap between these two estimates around the switching moment. Nevertheless, the method itself is continuous in *n* and does not contain any explicit switching rules. Therefore, there should be some continuous convergence rate estimate for our method, and it is an open question to find it.

Another interesting question is to understand what happens with the proposed method on other (less general) classes of convex problems than those, considered in this paper. For example, it is well-known that, on smooth and/or strongly convex problems, (sub)gradient methods have much better convergence rates than on the general nonsmooth problems. We expect that similar conclusions should also be valid for the proposed Subgradient Ellipsoid Method. However, to achieve the acceleration, it may be necessary to introduce some modifications in the algorithm such as using different step sizes. We leave this direction for future research.

Finally, apart from the Ellipsoid Method, there exist other “dimension-dependent” methods (e.g., the Center-of-Gravity Method^8^ [13, 20], the Inscribed Ellipsoid Method [22], the Circumscribed Simplex Method [6], etc.). Similarly, the Subgradient Method is not the only “dimension-independent” method and there exist numerous alternatives which are better suited for certain problem classes (e.g., the Fast Gradient Method [17] for Smooth Convex Optimization or methods for Stochastic Programming [7, 8, 11, 15]). Of course, it is interesting to consider different combinations of the aforementioned “dimension-dependent” and “dimension-independent” methods. In this regard, it is also worth mentioning the works [4, 5], where the authors propose new variants of gradient-type methods for smooth strongly convex minimization problems inspired by the geometric construction of the Ellipsoid Method.

## Proof of Lemma 6

### Proof

Everywhere in the proof, we assume that the parameter *c* is fixed and drop all the indices related to it.

Let us show that $\zeta _p$ is a convex function. Indeed, the function $\omega :\mathbb {R}\times \mathbb {R}_{++} \rightarrow \mathbb {R}$, defined by $\omega (x, t) :=\frac{x^2}{t}$, is convex. Hence, the function *q*, defined in (34), is also convex. Further, since $\omega$ is increasing in its first argument on $\mathbb {R}_+$, the function $\omega _p :\mathbb {R}_+\times \mathbb {R}_{++} \rightarrow \mathbb {R}$, defined by $\omega _p(x, t) :=\frac{x^p}{t}$, is also convex as the composition of $\omega$ with the mapping $(x, t) \mapsto (x^{p/2}, t)$, whose first component is convex (since $p \ge 2$) and the second one is affine. Note that $\omega _p$ is increasing in its first argument. Hence, $\zeta _p$ is indeed a convex function as the composition of $\omega _p$ with the mapping $\gamma \mapsto \bigl (q(\gamma ), 1 + \gamma \bigr )$, whose first part is convex and the second one is affine.

Differentiating, for any $\gamma > 0$, we obtain

$$\begin{aligned} \zeta _p'(\gamma ) {=} \frac{ p [q(\gamma )]^{p-1} q'(\gamma ) (1 + \gamma ) - [q(\gamma )]^p }{(1 + \gamma )^2} {=} \frac{ [q(\gamma )]^{p-1} \bigl ( p q'(\gamma ) (1 + \gamma ) - q(\gamma ) \bigr )}{(1 + \gamma )^2}. \end{aligned}$$

Therefore, the minimizers of $\zeta _p$ are exactly solutions to the following equation:

67 $$\begin{aligned} p q'(\gamma ) (1 + \gamma ) = q(\gamma ). \end{aligned}$$

Note that $q'(\gamma ) = \frac{c [2 \gamma (1 + \gamma ) - \gamma ^2]}{2 (1 + \gamma )^2} = \frac{c \gamma (2 + \gamma )}{2 (1 + \gamma )^2}$ (see (34)). Hence, (67) can be written as $c p \gamma (2 + \gamma ) = 2 (1 + \gamma ) + c \gamma ^2$ or, equivalently, $c (p - 1) \gamma ^2 + 2 (c p - 1) \gamma = 2$. Clearly, $\gamma = 0$ is not a solution of this equation. Making the change of variables $\gamma = \frac{2}{u}$, $u \ne 0$, we come the quadratic equation $u^2 - 2 (c p - 1) u = 2 c (p - 1)$ or, equivalently, to $[ u - (c p - 1) ]^2 = 2 c (p - 1) + (c p - 1)^2 = c^2 p^2 - (2 c - 1)$. This equation has two solutions: $u_1 :=c p - 1 + \sqrt{c^2 p^2 - (2 c - 1)}$ and $u_2 :=c p - 1 - \sqrt{c^2 p^2 - (2 c - 1)}$. Note that $u_2 \ge c p - 1 - \sqrt{c^2 p^2 + 1} \ge c p - 1 - (c p + 1) = -2$. Hence, $\gamma _2 :=\frac{2}{u_2} \le -1$ cannot be a minimizer of $\zeta _p$. Consequently, only $u_1$ is an acceptable solution (note that $u_1 > 0$ in view of our assumptions on *c* and *p*). Thus, (37) is proved.

Let us show that $\gamma (p)$ belongs to the interval specified in (37). For this, we need to prove that $1 \le c p \gamma (p) \le 2$. Note that the function $h_a(t) :=\frac{t}{\sqrt{t^2 - a} + t - 1}$, where $a \ge 0$, is decreasing in *t*. Indeed, $\frac{1}{h_a(t)} = \sqrt{1 - \frac{a}{t^2}} - \frac{1}{t} + 1$ is an increasing function in *t*. Hence, $c p \gamma (p) = 2 h_{2 c - 1}(c p) \ge 2 \lim _{t \rightarrow \infty } h_{2 c - 1}(t) = 1$. On the other hand, using that $p \ge 2$ and denoting $\alpha :=2 c \ge 1$, we get $c p \gamma (p) = 2 h_{\alpha - 1}(c p) \le 2 g(\alpha )$, where $g(\alpha ) :=h_{\alpha - 1}(\alpha ) = \frac{\alpha }{\sqrt{\alpha ^2 - \alpha + 1} + \alpha - 1}$. Note that *g* is decreasing in $\alpha$. Indeed, denoting $\tau :=\frac{1}{\alpha } \in \left( {0, 1} \right]$, we obtain $\frac{1}{g(\alpha )} = \sqrt{1 - \tau + \tau ^2} - \tau + 1$, which is a decreasing function in $\tau$. Thus, $c p \gamma (p) \le 2 g(1) = 2$.

It remains to prove that $\zeta _p(\gamma (p)) \le e^{-1 / (2 c p)}$. Let $\phi :\left[ {2, +\infty } \right) \rightarrow \mathbb {R}$ be the function

68 $$\begin{aligned} \phi (p) :=-\ln \zeta _p\bigl ( \gamma (p) \bigr ) = \ln \bigl ( 1 + \gamma (p) \bigr ) - p \ln q\bigl ( \gamma (p) \bigr ). \end{aligned}$$

We need to show that $\phi (p) \ge 1 / (2 c p)$ for all $p \ge 2$ or, equivalently, that the function $\chi :\left( {0, \frac{1}{2}} \right] \rightarrow \mathbb {R}$, defined by $\chi (\tau ) :=\phi (\frac{1}{\tau })$, satisfies $\chi (\tau ) \ge \frac{\tau }{2 c}$ for all $\tau \in \left( {0, \frac{1}{2}} \right]$. For this, it suffices to show that $\chi$ is convex, $\lim _{\tau \rightarrow 0} \chi (\tau ) = 0$ and $\lim _{\tau \rightarrow 0} \chi '(\tau ) = \frac{1}{2 c}$. Differentiating, we see that $\chi '(\tau ) = -\frac{1}{\tau ^2} \phi '(\frac{1}{\tau } )$ and $\chi ''(\tau ) = \frac{2}{\tau ^3} \phi '(\frac{1}{\tau } ) + \frac{1}{\tau ^4} \phi ''(\frac{1}{\tau } )$ for all $\tau \in \left( {0, \frac{1}{2}} \right]$. Thus, we need to justify that

69 $$\begin{aligned} 2 \phi '(p) + p \phi ''(p) \ge 0 \end{aligned}$$

for all $p \ge 2$ and that

70 $$\begin{aligned} \lim _{p \rightarrow \infty } \phi (p) = 0, \qquad \lim _{p \rightarrow \infty } [-p^2 \phi '(p)] = \frac{1}{2 c}. \end{aligned}$$

Let $p \ge 2$ be arbitrary. Differentiating and using (67), we obtain

71 $$\begin{aligned} \phi '(p)&= \frac{\gamma '(p)}{1 + \gamma (p)} - \ln q\bigl ( \gamma (p) \bigr ) - \frac{p q'\bigl ( \gamma (p) \bigr ) \gamma '(p)}{ q\bigl ( \gamma (p) \bigr )} = -\ln q\bigl ( \gamma (p) \bigr ),\nonumber \\ \phi ''(p)&= -\frac{q'(\gamma (p)) \gamma '(p)}{q(\gamma (p))} = -\frac{\gamma '(p)}{p \bigl ( 1 + \gamma (p) \bigr )}. \end{aligned}$$

Therefore,

$$\begin{aligned} 2 \phi '(p) + p \phi ''(p) = -2 \ln q(\gamma (p)) - \frac{\gamma '(p)}{1 + \gamma (p)} \ge -\frac{c \gamma ^2(p) + \gamma '(p)}{1 + \gamma (p)}, \end{aligned}$$

where the inequality follows from (34) and the fact that $\ln (1 + \tau ) \le \tau$ for any $\tau > -1$. Thus, to show (69), we need to prove that $-\gamma '(p) \ge c \gamma ^2(p)$ or, equivalently, $\frac{d}{d p} \frac{1}{\gamma (p)} \ge c$. But this is immediate. Indeed, using (37), we obtain $\frac{d}{d p} \frac{1}{\gamma (p)} = \frac{c}{2} \left( \frac{c p}{\sqrt{c^2 p^2 - (2 c - 1)}} + 1 \right) \ge c$ since the function $\tau \mapsto \frac{\tau }{\sqrt{\tau ^2 - 1}}$ is decreasing. Thus, (69) is proved.

It remains to show (70). From (37), we see that $\gamma (p) \rightarrow 0$ and $p \gamma (p) \rightarrow \frac{1}{c}$ as $p \rightarrow \infty$. Hence, using (34), we obtain

$$\begin{aligned} \lim _{p \rightarrow \infty } p^2 \ln q\bigl ( \gamma (p) \bigr ) = \lim _{p \rightarrow \infty } \frac{c p^2 \gamma ^2(p)}{ 2 \bigl ( 1 + \gamma (p) \bigr )} = \frac{c}{2} \lim _{p \rightarrow \infty } p^2 \gamma ^2(p) = \frac{1}{2 c}. \end{aligned}$$

Consequently, in view of (68) and (71), we have

$$\begin{aligned} \begin{aligned} \lim _{p \rightarrow \infty } \phi (p)&= \lim _{p \rightarrow \infty } \bigl [ \ln \bigl ( 1 + \gamma (p) \bigr ) - p \ln q\bigl ( \gamma (p) \bigr ) \bigr ] = 0, \\ \lim _{p \rightarrow \infty } [-p^2 \phi '(p)]&= \lim _{p \rightarrow \infty } p^2 \ln q\bigl ( \gamma (p) \bigr ) = \frac{1}{2 c}, \end{aligned} \end{aligned}$$

which is exactly (70). $\square$

## Support function and dual multipliers: proofs

For brevity, everywhere in this section, we write $\Vert x \Vert$ and ${\Vert {\cdot } \Vert }_*$ instead of ${\Vert \cdot \Vert }_{H^{-1}}$ and ${\Vert \cdot \Vert }_{H^{-1}}^*$, respectively. We also denote $B_0 :=\lbrace {x \in \mathbb {E} : \Vert x \Vert \le 1} \rbrace$.

### Auxiliary operations

#### Lemma 9

Let $s \in \mathbb {E}^*$, let $A :\mathbb {R}^m \rightarrow \mathbb {E}^*$ be a linear operator with trivial kernel and let $b \in \mathbb {R}^m$, $\langle {b, (A^*H A)^{-1} b} \rangle < 1$. Then, problem (62) has a unique solution given by (63).

#### Proof

Note that the sublevel sets of the objective function in (62) are bounded:

$$\begin{aligned} {\Vert {s {-} A u} \Vert }_* {+} \langle {u, b} \rangle \ge {\Vert {A u} \Vert }_* {-} {\Vert {s} \Vert }_* {+} \langle {u, b} \rangle \ge (1 {-} \langle {b, (A^*H A)^{-1} b} \rangle ^{1/2}) {\Vert {A u} \Vert }_* {-} {\Vert {s} \Vert }_* \end{aligned}$$

for all $u \in \mathbb {R}^m$. Hence, problem (62) has a solution.

Let $u \in \mathbb {R}^m$ be a solution of problem (62). If $s = A u$, then $u = (A^*H A)^{-1} A^*s$, which coincides with the solution given by (63) (note that, in this case, $r = 0$).

Now suppose $s \ne A u$. Then, from the first-order optimality condition, we obtain that $b = A^*(s - A u) / \rho$, where $\rho :={\Vert {s - A u} \Vert }_* > 0$. Hence, $u = (A^*H A)^{-1} (A^*s - \rho b)$ and

$$\begin{aligned} \begin{aligned} \rho ^2&= {\Vert {s - A u} \Vert }_*^2 = {\Vert {s} \Vert }_*^2 - 2 \langle {A^*s, u} \rangle + \langle {A^*H A u, u} \rangle \\&= {\Vert {s} \Vert }_*^2 - 2 \langle {A^*s, (A^*H A)^{-1} (A^*s - \rho b)} \rangle + \langle {A^*s - \rho b, (A^*H A)^{-1} (A^*s - \rho b)} \rangle \\&= {\Vert {s} \Vert }_*^2 - \langle {s, A (A^*H A)^{-1} A^*s} \rangle + \rho ^2 \langle {b, (A^*H A)^{-1} b} \rangle . \end{aligned} \end{aligned}$$

Thus, $\rho = r$ and $u = u(H, s, A, b)$ given by (63). $\square$

#### Lemma 10

Let $s, a \in \mathbb {E}^*$, $\beta \in \mathbb {R}$ be such that $\langle {a, x} \rangle \le \beta$ for some $x \in {{\,\mathrm{int}\,}}B_0$. Then, problem (60) has a solution given by (61). Moreover, this solution is unique if $\beta < {\Vert {a} \Vert }_*$.

#### Proof

Let $\phi :\mathbb {R} \rightarrow \mathbb {R}$ be the function $\phi (\tau ) :={\Vert {s - \tau a} \Vert }_* + \tau \beta$. By our assumptions, $\beta > -{\Vert {a} \Vert }_*$ if $a \ne 0$ and $\beta \ge 0$ if $a = 0$. If additionally $\beta < {\Vert {a} \Vert }_*$, then $|\beta | < {\Vert {a} \Vert }_*$.

If $s = 0$, then $\phi (\tau ) = \tau ({\Vert {a} \Vert }_* + \beta ) \ge \phi (0)$ for all $\tau \ge 0$, so 0 is a solution of (60). Clearly, this solution is unique when $\beta < {\Vert {a} \Vert }_*$ because then $|\beta | < {\Vert {a} \Vert }_*$.

From now on, suppose $s \ne 0$. Then, $\phi$ is differentiable at 0 with $\phi '(0) = \beta - \langle {a, s} \rangle / {\Vert {s} \Vert }_*$. If $\langle {a, s} \rangle \le \beta {\Vert {s} \Vert }_*$, then $\phi '(0) \ge 0$, so 0 is a solution of (60). Note that this solution is unique if $\langle {a, s} \rangle < \beta {\Vert {s} \Vert }_*$ because then $\phi '(0) > 0$, i.e., $\phi$ is strictly increasing on $\mathbb {R}_+$.

Suppose $\langle {a, s} \rangle > \beta {\Vert {s} \Vert }_*$. Then, $\beta < {\Vert {a} \Vert }_*$ and thus $|\beta | < {\Vert {a} \Vert }_*$. Note that, for any $\tau \ge 0$, we have $\phi (\tau ) \ge \tau ({\Vert {a} \Vert }_* + \beta ) - {\Vert {s} \Vert }_*$. Hence, the sublevel sets of $\phi$, intersected with $\mathbb {R}_+$, are bounded, so problem (60) has a solution. Since $\phi '(0) < 0$, any solution of (60) is strictly positive and so must be a solution of problem (62) for $A :=a$ and $b :=\beta$. But, by Lemma 9, the latter solution is unique and equals $u(H, s, a, \beta )$.

We have proved that (61) is indeed a solution of (60). Moreover, when $\langle {a, s} \rangle \ne \beta {\Vert {s} \Vert }_*$, we have shown that this solution is unique. It remains to prove the uniqueness of solution when $\langle {a, s} \rangle = \beta {\Vert {s} \Vert }_*$, assuming additionally that $\beta < {\Vert {a} \Vert }_*$. But this is simple. Indeed, by our assumptions, $|\beta | < {\Vert {a} \Vert }_*$, so $|\langle {a, s} \rangle | = |\beta | {\Vert {s} \Vert }_* < {\Vert {a} \Vert }_* {\Vert {s} \Vert }_*$. Hence, *a* and *s* are linearly independent. But then $\phi$ is strictly convex, and thus its minimizer is unique. $\square$

### Computation of dual multipliers

In this section, we prove the correctness of Algorithm 3.

For $s \in \mathbb {E}^*$, let *X*(*s*) be the subdifferential of ${\Vert {\cdot } \Vert }_*$ at the point *s*:

72 $$\begin{aligned} X(s) :={\left\{ \begin{array}{ll} \lbrace H s / {\Vert {s} \Vert }_*\rbrace , &{} \text {if}\, s \ne 0, \\ B_0, &{} \text {if}\, s = 0. \end{array}\right. } \end{aligned}$$

Clearly, $X(s) \subseteq B_0$ for any $s \in \mathbb {E}^*$. When $s \ne 0$, we denote the unique element of *X*(*s*) by *x*(*s*).

Let us formulate a convenient optimality condition.

#### Lemma 11

Let *A* be the linear operator from $\mathbb {R}^m$ to $\mathbb {E}^*$, defined by $A u :=\sum _{i=1}^m u_i a_i$, where $a_1, \dots , a_m \in \mathbb {E}^*$, and let $b \in \mathbb {R}^m$, $s \in \mathbb {E}^*$. Then, $\mu ^* \in \mathbb {R}_+^m$ is a minimizer of $\psi (\mu ) :={\Vert {s - A \mu } \Vert }_* + \langle {\mu , b} \rangle$ over $\mathbb {R}_+^m$ if and only if $X(s - A \mu ^*) \cap L_1(\mu _1^*) \dots L_m(\mu _m^*) \ne \emptyset$, where, for each $1 \le i \le m$ and $\tau > 0$, we denote $L_i(\tau ) :=\lbrace {x \in \mathbb {E} : \langle {a_i, x} \rangle \le b_i} \rbrace$, if $\tau = 0$, and $L_i(\tau ) :=\lbrace {x \in \mathbb {E} : \langle {a_i, x} \rangle = b_i} \rbrace$, if $\tau > 0$.

#### Proof

Indeed, the standard optimality condition for a convex function over the nonnegative orthant is as follows: $\mu ^* \in \mathbb {R}_+^m$ is a minimizer of $\psi$ on $\mathbb {R}_+^m$ if and only if there exists $g^* \in \partial \psi (\mu ^*)$ such that $g_i^* \ge 0$ and $g_i^* \mu _i^* = 0$ for all $1 \le i \le m$. It remains to note that $\partial \psi (\mu ^*) = b - A^*X(s - A \mu ^*)$. $\square$

#### Theorem 3

Algorithm 3 is well-defined and returns a solution of (65).

#### Proof

i. For each $i = 1, 2$ and $\tau \ge 0$, denote $L_i^- :=\lbrace {x \in \mathbb {E} : \langle {a_i, x} \rangle \le b_i} \rbrace$, $L_i :=\lbrace {x \in \mathbb {E} : \langle {a_i, x} \rangle = b_i} \rbrace$, $L_i(\tau ) :=L_i^-$, if $\tau = 0$, and $L_i(\tau ) :=L_i$, if $\tau > 0$.

ii. From (66) and Lemma 10, it follows that Step  is well-defined and, for each $i = 1, 2$, $\tau _i$ is a solution of (60) with parameters $(s, a_i, b_i)$. Hence, by Lemma 11,

73 $$\begin{aligned} X(s - \tau _i a_i) \cap L_i(\tau _i) \ne \emptyset , \quad i = 1, 2. \end{aligned}$$

iii. Consider Step . Note that the condition $\xi _1 \le b_2$ is equivalent to $B_0 \cap L_1^- \subseteq L_2^-$ since $\xi _1 = \max _{x \in B_0 \cap L_1^-} \langle {a_2, x} \rangle$. If $B_0 \cap L_1^- \subseteq L_2^-$, then, by (73), $X(s - \tau _1 a_1) \cap L_1(\tau _1) \cap L_2^- = X(s - \tau _1 a_1) \cap L_1(\tau _1) \ne \emptyset$, so, by Lemma 11, $(\tau _1, 0)$ is indeed a solution of (65).

Similarly, if $\xi _2 \le b_1$, then $B_0 \cap L_2^- \subseteq L_1^-$ and $(0, \tau _2)$ is a solution of (65).

iv. From now on, we can assume that $B_0 \cap L_1^- \cap {{\,\mathrm{int}\,}}L_2^+ \ne \emptyset$, $B_0 \cap L_2^- \cap {{\,\mathrm{int}\,}}L_1^+ \ne \emptyset$, where ${{\,\mathrm{int}\,}}L_i^+ :=\lbrace {x \in \mathbb {E} : \langle {a_i, x} \rangle > b_i} \rbrace$, $i = 1, 2$. Combining this with (66), we obtain^9^

74 $$\begin{aligned} {{\,\mathrm{int}\,}}B_0 \cap L_1 \cap L_2^- \ne \emptyset , \qquad {{\,\mathrm{int}\,}}B_0 \cap L_2 \cap L_1^- \ne \emptyset . \end{aligned}$$

Suppose $\langle {a_2, H (s - \tau _1 a_1)} \rangle \le b_2 {\Vert {s - \tau _1 a_1} \Vert }_*$ at Step . 1) If $s \ne \tau _1 a_1$, then $X(s - \tau _1 a_1)$ is a singleton, $x(s - \tau _1 a_1) = H (s - \tau _1 a_1) / {\Vert {s - \tau _1 a_1} \Vert }_*$, so we obtain $x(s - \tau _1 a_1) \in L_2^-$. Combining this with (73), we get $x(s - \tau _1 a_1) \in L_1(\tau _1) \cap L_2^-$. 2) If $s = \tau _1 a_1$, then $X(s - \tau _1 a_1) \cap L_1(\tau _1) \cap L_2^- = B_0 \cap L_1(\tau _1) \cap L_2^- \ne \emptyset$ in view of the first claim in (74) (recall that $L_1 \subseteq L_1(\tau _1)$). Thus, in any case, $X(s - \tau a_1) \cap L_1(\tau _1) \cap L_2^- \ne \emptyset$, and so, by Lemma 11, $(\tau _1, 0)$ is a solution of (65).

Similarly, one can consider the case when $\langle {a_1, H (s - \tau _2 a_2)} \rangle \le b_1 {\Vert {s - \tau _2 a_2} \Vert }_*$ at Step .

Suppose we have reached Step . From now on, we can assume that

75 $$\begin{aligned} X(s - \tau _1 a_1) \cap L_1(\tau _1) \cap {{\,\mathrm{int}\,}}L_2^+ \ne \emptyset , \qquad X(s - \tau _2 a_2) \cap L_2(\tau _2) \cap {{\,\mathrm{int}\,}}L_1^+ \ne \emptyset .\nonumber \\ \end{aligned}$$

Indeed, since both conditions at Step  have not been satisfied, $s \ne \tau _i a_i$, $i = 1, 2$, and $x(s - \tau _1 a_1) \notin L_2^-$, $x(s - \tau _2 a_2) \notin L_1^-$. Also, by (73), $x(s - \tau _i a_i) \in L_i(\tau _i)$, $i = 1, 2$.

Let $\mu \in \mathbb {R}_+^2$ be any solution of (65). By Lemma 11, $X(s - A \mu ) \cap L_1(\mu _1) \cap L_2(\mu _2) \ne \emptyset$. Note that we cannot have $\mu _2 = 0$. Indeed, otherwise, we get $X(s - \mu _1 a_1) \cap L_1(\mu _1) \cap L_2^- \ne \emptyset$, so $\mu _1$ must be a solution of (60) with parameters $(s, a_1, b_1)$. But, by Lemma 10, such a solution is unique (in view of the second claim in (75), $\langle {a_1, x} \rangle > b_1$ for some $x \in B_0$, so $b_1 < {\Vert {a_1} \Vert }_*$). Hence, $\mu _1 = \tau _1$, and we obtain a contradiction with (75). Similarly, we can show that $\mu _1 \ne 0$. Consequently, $\mu _1, \mu _2 > 0$, which means that $\mu$ is a solution of (62).

Thus, at this point, any solution of (65) must be a solution of (62). In view of Lemma 9, to finish the proof, it remains to show that the vectors $a_1$, $a_2$ are linearly independent and $\langle {b, (A^*H A)^{-1} b} \rangle < 1$. But this is simple. Indeed, from (75), it follows that

76 $$\begin{aligned} \text {either} \quad B_0 \cap L_1 \cap {{\,\mathrm{int}\,}}L_2^+ \ne \emptyset \quad \text {or} \quad B_0 \cap L_2 \cap {{\,\mathrm{int}\,}}L_1^+ \ne \emptyset \end{aligned}$$

since $\tau _1$ and $\tau _2$ cannot both be equal to 0. Combining (76) and (74), we see that ${{\,\mathrm{int}\,}}B_0 \cap L_1 \cap L_2 \ne \emptyset$ and, in particular, $L_1 \cap L_2 \ne \emptyset$. Hence, $a_1$, $a_2$ are linearly independent (otherwise, $L_1 = L_2$, which contradicts (76)). Taking any $x \in {{\,\mathrm{int}\,}}B_0 \cap L_1 \cap L_2$, we obtain $\Vert x \Vert < 1$ and $A^*x = b$, hence $\langle {b, (A^*H A)^{-1} b} \rangle = \langle {A^*x, (A^*H A)^{-1} A^*x} \rangle \le \Vert x \Vert ^2 < 1$, where we have used $A (A^*H A)^{-1} A^*\preceq H^{-1}$. $\square$

## Footnotes

1 More precisely, $g_k$ must be a non-zero vector such that $\langle {g_k, x_k - x} \rangle \ge 0$ for all $x \in Q$. In particular, for the Euclidean ball, one can take $g_k :=x_k$.

2 Hereinafter, a *solid* is any convex compact set with nonempty interior.

3 In fact, we have proved the second estimate in (45) for all $k \ge 1$ (not only for $k \ge n^2$).

4 Recall that $g_i \ne 0$ for all $i \ge 0$ by (2).

5 Here eigenvectors and eigenvalues are defined with respect to the operator *B* inducing the norm $\Vert x \Vert$.

6 Note that, in (2), we identify the spaces $\mathbb {E}$, $\mathbb {E}^*$ with $\mathbb {R}^n$ in such a way that $\langle {\cdot , \cdot } \rangle$ coincides with the standard dot-product and $\Vert x \Vert$ coincides with the standard Euclidean norm. Therefore, *B* becomes the identity matrix and $g_k^*$ becomes $g_k^T$.

7 Hereinafter, we identify $(\mathbb {R}^m)^*$ with $\mathbb {R}^m$ in such a way that $\langle {\cdot , \cdot } \rangle$ is the standard dot product.

8 Although this method is not practical, it is still interested from an academic point of view.
